# Mesoporous Ag_2_Se as a model n-type thermoelectric: soft-templated synthesis and thermal transport engineering

**DOI:** 10.1039/d6sc05923f

**Published:** 2026-10-13

**Authors:** Zihao Yang, Minsu Han, Jihyun Kim, Ho Ngoc Nam, Quan Manh Phung, Young Jun Lee, Norman C.-R. Chen, Xiangyang Liu, Yuqi Guo, Yingji Zhao, Yunqing Kang, Kevin C.-W. Wu, Yusuke Asakura, Byeonggwan Kim, Yusuke Yamauchi

**Affiliations:** a Department of Materials Process Engineering, Graduate School of Engineering, Nagoya University Nagoya 464-8603 Japan minsu.han@nagoya-u.jp y.yamauchi@uq.edu.au; b Australian Institute for Bioengineering and Nanotechnology (AIBN), The University of Queensland Brisbane QLD 4072 Australia; c Department of Chemical Engineering and Applied Chemistry, Chungnam National University 99 Daehak-ro, Yuseong-gu Daejeon 34134 Republic of Korea; d Department of Chemistry, Graduate School of Science, Nagoya University Nagoya 464-8602 Japan; e Institute of Transformative Bio-Molecules (WPI-ITbM), Nagoya University Nagoya 464-8601 Japan; f Carbon Composite Materials Research Center, Korea Institute of Science and Technology (KIST) 92 Chudong-ro, Bongdong-eup Wanju-gun Jeollabuk-do 55324 Republic of Korea; g Molecular Science and Technology Program, Taiwan International Graduate Program, Academia Sinica Taipei 10617 Taiwan; h International Graduate Program of Molecular Science and Technology (NTU-MST), National Taiwan University Taipei 10617 Taiwan; i Department of Chemical Engineering, National Taiwan University Taipei 10617 Taiwan; j Center of Atomic Initiative for New Materials (AI-MAT), National Taiwan University Taipei 10617 Taiwan; k Department of Chemical and Biomolecular Engineering, Yonsei University 50 Yonsei-ro, Seodaemun-gu Seoul 03722 Republic of Korea

## Abstract

Mesoporous structuring is a promising route to suppress thermal conductivity in thermoelectrics by enhancing phonon scattering, yet mesoporous thermoelectric materials remain scarcely explored. Here, we report the synthesis and thermoelectric evaluation of mesoporous Ag_2_Se as a model n-type system. Mesoporous Ag_2_Se thin films are obtained by *block*-copolymer-directed soft-templated electrodeposition using PS-*b*-PEO micelles, which interact with Ag^+^ and Se(iv) species and direct the formation of orthorhombic β-Ag_2_Se frameworks with a high density of mesopores. By tuning the molecular weight of copolymers and applying an optimized deposition potential of −0.48 V *vs.* Hg/Hg_2_SO_4_ (saturated K_2_SO_4_), we realize narrowly distributed pore diameters of 8, 12, and 16 nm and a concomitant deposition in crystallite size. The films are delaminated from ITO substrates and pressed into pellets while preserving both the mesoporous architecture and the β-Ag_2_Se lattice. For the Ag_2_Se-12 nm sample, the thermal conductivity at room temperature is reduced from 0.93 W m^−1^ K^−1^ for nonporous Ag_2_Se to 0.40 W m^−1^ K^−1^, with moderate degradation of the power factor, resulting in a room-temperature *zT* of 0.98 (56% enhancement). These results establish mesostructural engineering in Ag_2_Se as an effective strategy to reduce the thermal conductivity, providing a design principle for next-generation thermoelectrics.

## Abstract 2

Soft-templated mesostructuring of Ag_2_Se enhances phonon scattering and markedly suppresses thermal transport. The engineered nanoscale architecture produces a substantial reduction in thermal conductivity across the measured temperature range.

## Graphical abstract

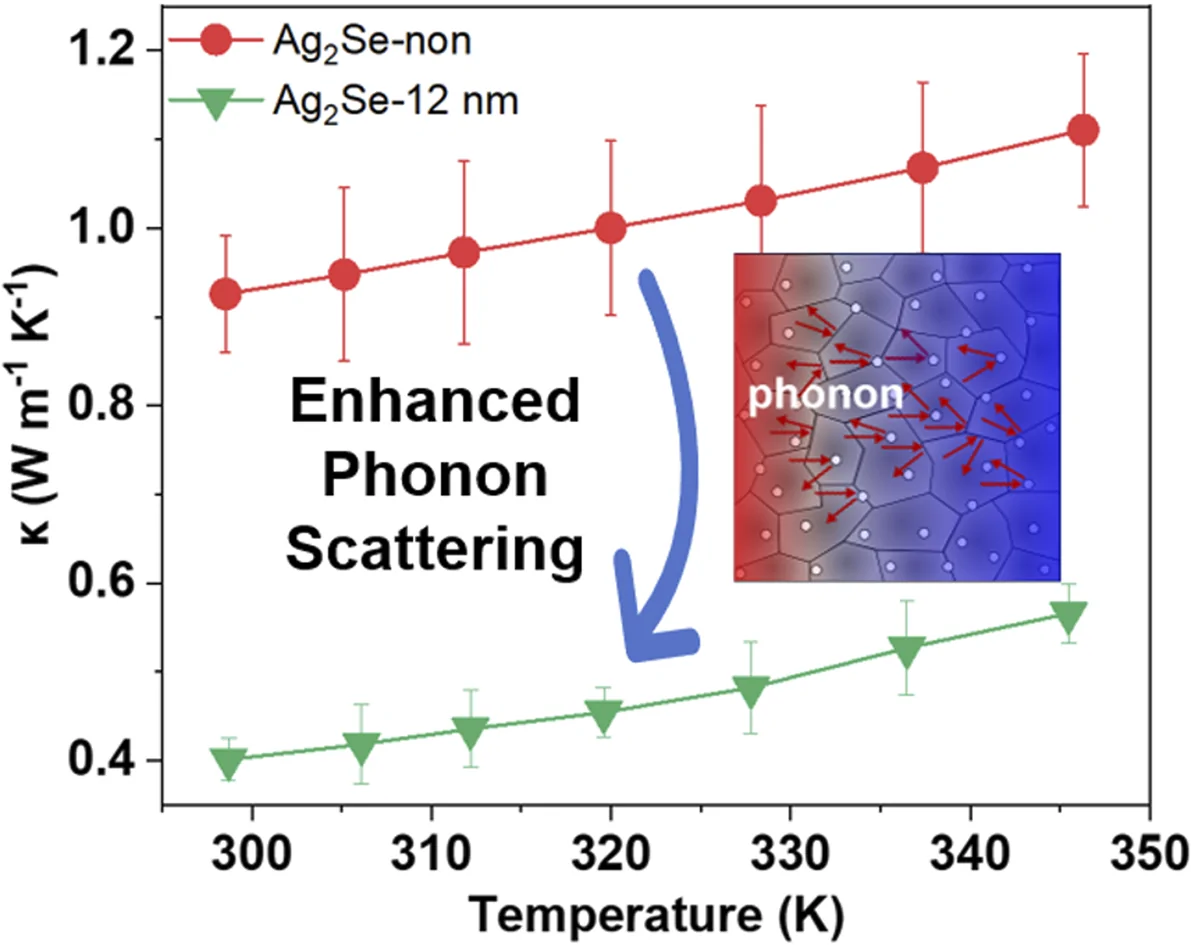

## Introduction

Thermoelectric energy conversion offers a compelling route to recover the vast amounts of low-grade heat that accompany modern energy use.^1^ A large fraction of primary energy input in industrial processes, transportation, and electronic devices is ultimately dissipated as waste heat, which is typically rejected to the environment through heat exchangers and radiative loss, thereby limiting overall system efficiency. Efficiently converting even a modest portion of this ubiquitous heat flux into electricity would alleviate the growing energy demand and reduce associated greenhouse-gas emissions.^2–4^ Against this backdrop, solid-state thermoelectric materials are uniquely attractive because they can convert heat directly into electrical power without moving parts, working fluids, or noise, and can operate across a broad range of length scales—from chip-level coolers to large-area generators.^5–8^

The performance of thermoelectric materials is quantified by the dimensionless figure of merit (*zT*),
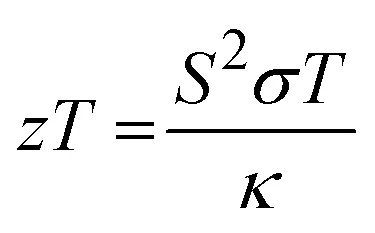
where *S* is the Seebeck coefficient, *σ* is the electrical conductivity, *T* is the absolute temperature, and *κ* is the total thermal conductivity. The latter is the sum of electronic (*κ*_e_) and lattice contributions (*κ*_L_), *κ* = *κ*_e_ + *κ*_L_.^9^ High efficiency requires a large power factor (PF), *S*^2^*σ*, combined with a low *κ*. However, these transport parameters are strongly interdependent through the carrier concentration, effective mass, and scattering mechanisms.^10–15^ In conventional semiconducting thermoelectrics, increasing the effective mass enhances *S* but typically suppresses *σ*,^16^ and the Wiedemann–Franz relation further links *σ* to *κ*_e_.^17^ As a result, strategies based solely on PF optimization *via* heteroatom doping, composition tuning, or band-structure engineering can only deliver limited gains once the optimum carrier concentration is approached.^18–20^

An alternative and often more powerful route to increasing *zT* is to selectively suppress *κ*, while preserving favorable electronic transport.^21^ Because most state-of-the-art thermoelectric materials are heavily doped semiconductors in which the lattice contribution *κ*_L_ constitutes a major fraction of *κ*, the key design challenge is to maximize phonon scattering without unduly perturbing the charge transport.^22,23^ Structural approaches spanning multiple length scales have been pursued, including control of crystallinity and grain size,^24^ alloying-induced point defects,^25^ second-phase nanoprecipitates,^26^ and complex hierarchical architectures^27^ that combine several scattering mechanisms.

Engineering nanostructures is particularly attractive because phonon transport can be strongly perturbed while maintaining the intrinsic composition of the thermoelectric host.^28–30^ Nano- and microscale structural features can introduce additional phonon-scattering interfaces and shorten effective phonon transport lengths, thereby reducing thermal conductivity.^31,32^ Accordingly, various microstructural engineering strategies have recently been explored.^33,34^ For example, Guo *et al.* have demonstrated that nanostructured Ag_2_Se can be obtained by suppressing densification through reduced powder plasticity during consolidation.^35^ In another approach, extrusion-based 3D printing followed by pressure-free sintering produced Ag_2_Se with a highly porous microstructure and substantially reduced thermal conductivity.^36^ Despite these advances, systematic studies on the thermoelectric effects of well-defined nanoscale structures in compositionally pristine Ag_2_Se remain limited. Precise control over nanoscale feature size and spatial distribution has rarely been demonstrated.

Ag_2_Se is a leading n-type thermoelectric near room temperature, combining a narrow bandgap and high carrier mobility with intrinsically low *κ*_L_ that stems from its soft lattice and highly mobile Ag sublattice.^37,38^ Consequently, both bulk and thin-film Ag_2_Se can deliver high PFs and *zT* values comparable to those of conventional Bi_2_Te_3_-based alloys, while also offering compatibility with low-temperature, solution-based processing.^39–41^ Beyond its favorable transport properties, Ag_2_Se is attractive as a Te-free alternative to conventional Bi_2_Te_3_-based thermoelectrics.^42^ These attributes make Ag_2_Se particularly well-suited as a “host” in which thermal transport can be deliberately modulated through nanoscale structural engineering while avoiding intentional compositional modification. Kitthonbancha *et al.* recently employed a transient-liquid approach to obtain porous Ag_2_Se, reaching a low *κ* of 0.73 W m^−1^ K^−1^ and a room-temperature *zT* of 1.04.^43^ Yet, the resulting Ag_2_Se exhibits a highly random microstructure and contains Na_2_SO_3_ within the porous network, making it difficult to deconvolute the intrinsic effect of nanoporosity from compositional changes. To elucidate how pore architectures govern both thermal transport and overall thermoelectric performance, it is therefore essential to develop model thermoelectric materials that combine intrinsically good properties with well-controlled, reproducible pore architectures.

Herein, we develop mesoporous Ag_2_Se as a model thermoelectric platform in which the influence of mesoporous architecture on heat and charge transport can be elucidated. We demonstrate, for the first time, the synthesis of Ag_2_Se with uniformly distributed mesopores of 8–16 nm throughout the entire volume, realized *via block*-copolymer-directed soft-templated electrodeposition. By precisely controlling the reduction potential during electrodeposition, the Ag : Se stoichiometry and pore architecture are tuned simultaneously. The resulting mesoporous Ag_2_Se films grown on indium tin oxide (ITO) glasses are subsequently detached by ultrasonication, collected as powders, and cold-pressed into pellets for thermoelectric measurements. This low-temperature pelletization preserves the preformed mesostructure, thereby minimizing Ag-ion redistribution and the associated microstructural evolution of Ag_2_Se. Remarkably, despite a decrease in electrical conductivity and Seebeck coefficient, the introduction of mesopores lowers the room-temperature thermal conductivity from 0.93 for the nonporous sample to 0.40 W m^−1^ K^−1^, yielding an overall 56% enhancement in *zT*. We attribute this pronounced suppression of *κ* to the dense population of nanoscale pores, assisted by the reduced crystallite size resulting from *block*-copolymer micelle-templated growth, both of which enhance phonon scattering (Fig. 1). These results establish mesostructural engineering—specifically, the deliberate incorporation of well-defined pore networks—as a powerful design strategy for suppressing thermal transport, highlighting mesoporous thermoelectrics as a promising direction for next-generation high-efficiency materials for low-grade waste-heat recovery.

**Fig. 1:**
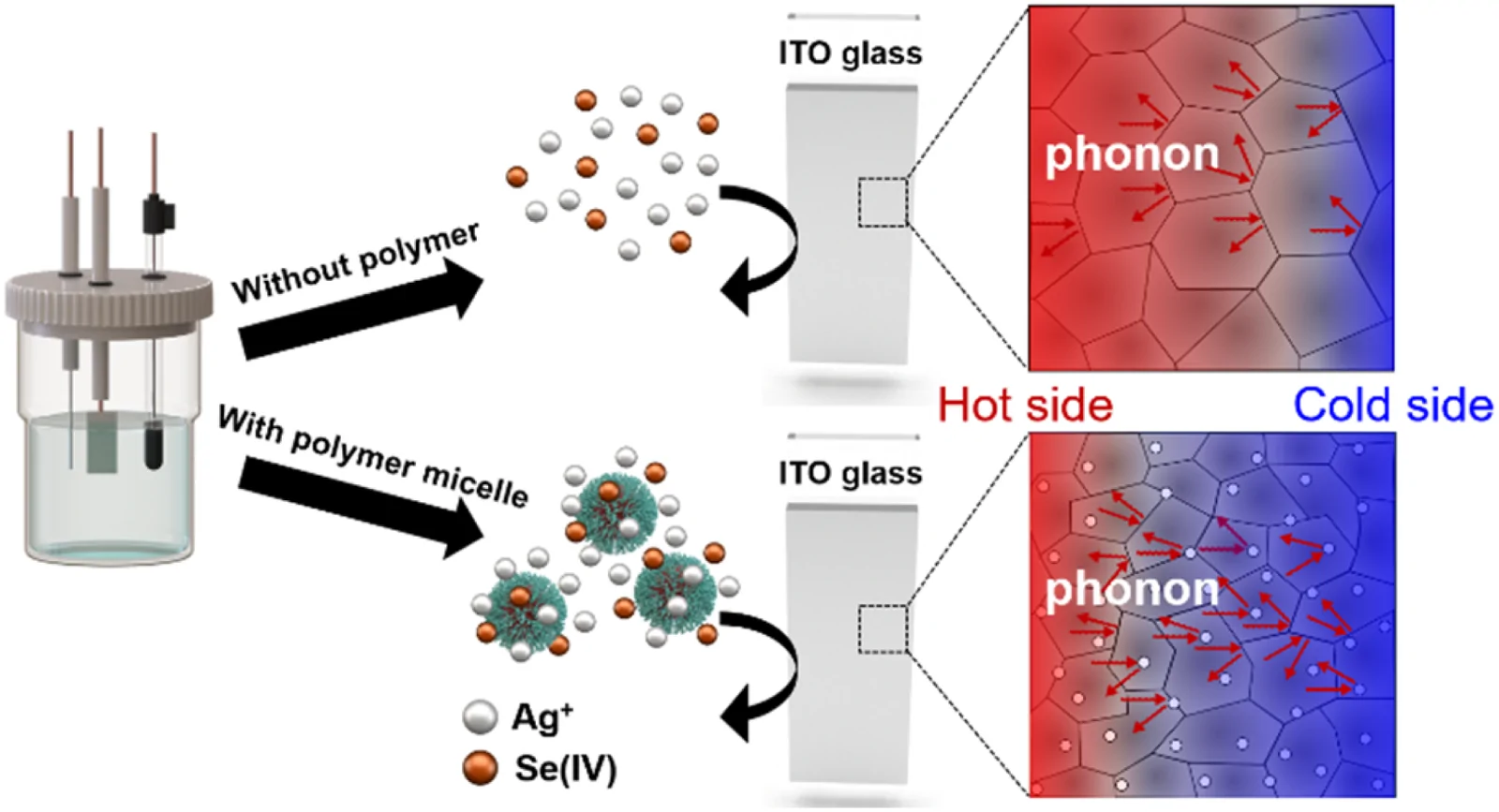
Schematic illustration of mesoporous Ag_2_Se synthesis *via* a *block*-copolymer-based soft-templating route and the resulting enhancement of phonon scattering in the mesoporous architecture.

## Results and discussion

### Interactions between PS-*b*-PEO micelles and precursor ions in synthetic solutions

Mesoporous Ag_2_Se thin films were prepared *via* a *block*-copolymer-directed soft-templating strategy.^44,45^ Polystyrene-*block*-poly(ethylene oxide) (PS-*b*-PEO) was first fully dissolved in *N*,*N*-dimethylformamide (DMF) to form a unimer solution, after which ethanol, 80 mM AgNO_3_, 3.0 M HNO_3_, and 80 mM SeO_2_ solutions were slowly added to this solution in sequence. The amphiphilic PS-*b*-PEO spontaneously self-assembles into spherical micelles with a hydrophobic PS core and a hydrophilic PEO corona when water is introduced into the system, driven by the unfavorable interaction between the PS block and water. Micelle formation is evidenced by the Tyndall effect, which becomes more pronounced upon addition of aqueous HNO_3_ to a DMF solution of PS-*b*-PEO (Fig. S1, SI). The Tyndall scattering is further enhanced after introducing the precursor ions (AgNO_3_ and SeO_2_), suggesting partial aggregation or growth of the micellar assemblies. By contrast, no appreciable light scattering is observed when the same precursor solution is added to a DMF solution without the *block* copolymer (Fig. S2), confirming that the Tyndall effect originates from micelles formed by PS-*b*-PEO. The intensity of the Tyndall effect is independent of the molecular weight of the *block* copolymer.

The interactions between PS-*b*-PEO micelles and precursor ions were investigated by electrophoretic light scattering (ELS) (Fig. 2). To obtain reliable zeta potential (*ζ*) values, ELS measurements were performed in a model electrolyte whose composition ratios matched those of the Ag_2_Se synthesis bath but with all solute concentrations reduced tenfold (see Table S1 for the detailed composition). In the DMF/ethanol/water mixture without added acid or inorganic precursors, the PS-*b*-PEO dispersion exhibits a nearly neutral *ζ* of −0.58 mV and a low ionic conductivity of 0.046 mS cm^−1^. Upon addition of 0.3 M HNO_3_, the ionic conductivity increases to 21.10 mS cm^−1^ owing to the higher concentration of mobile ions, and the *ζ* shifts to a more negative value (−8.62 mV). Further addition of 8 mM NaNO_3_ causes only a slight additional shift of the *ζ* (−10.32 mV), while the ionic conductivity remains largely unchanged because the ionic strength is already high. In contrast, addition of 8 mM AgNO_3_ drives a more pronounced shift in the *ζ* to −13.42 mV and is accompanied by a decrease in ionic conductivity. This behavior is consistent with coordination of Ag^+^ to the ether oxygen atoms of the PEO blocks,^46,47^ leading to preferential localization of Ag^+^ and associated NO_3_^−^ counterions within the PEO-rich micelle corona. Introducing 8 mM SeO_2_ results in a further increase in the magnitude of the negative *ζ* to −19.20 mV. We attribute this to specific interactions—most likely hydrogen bonding and coordination—between the PEO ether oxygens and Se(iv) species such as selenious acid (H_2_SeO_3_) and hydrogen selenite (HSeO_3_^−^), which are known to engage in strong hydrogen bonding with oxygen donors.^48,49^ In a solution containing both AgNO_3_ and SeO_2_, the *ζ* becomes less negative (−14.93 mV) than in the SeO_2_-only case. This trend may be associated with partial sequestration of Se(iv) into Ag–selenite species (*e.g.*, AgSeO_3_^−^),^50^ which reduces the amount of free Se(iv) available to interact with PEO. However, unambiguous identification of the ionic species and their coordination environments under these strongly acidic conditions would require further spectroscopic and speciation analyses. Overall, these trends indicate that PS-*b*-PEO micelles interact with both Ag^+^ and Se(iv) species in solution. When an appropriate cathodic potential is applied to an immersed electrode in this synthetic solution, Ag_2_Se is electrodeposited in intimate association with the polymer micelles.

**Fig. 2:**
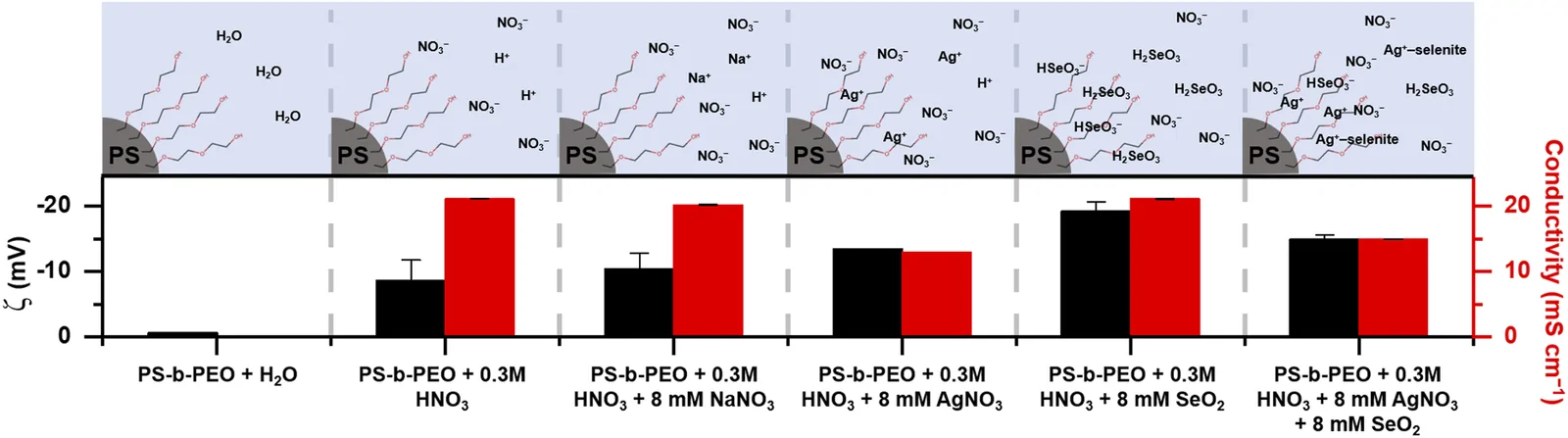
Schematic diagram (top) illustrating the proposed interaction model between polymer micelles and ions in each solution, together with the zeta potential and ionic conductivity (bottom) of solutions with different compositions.

### Electrodeposition of mesoporous Ag_2_Se and crystallographic analysis

When a cathodic potential is applied to an ITO substrate immersed in the mixed electrolyte, Ag^+^ and Se(iv) species are co-deposited at the electrode surface, leading to the formation of Ag_2_Se. Depending on the presence and molecular weight of the polymer in the deposition bath, the resulting films are denoted Ag_2_Se-non (polymer-free), Ag_2_Se-8 nm (PS_5,000_-*b*-PEO_2,500_), Ag_2_Se-12 nm (PS_9,000_-*b*-PEO_3,500_), and Ag_2_Se-16 nm (PS_19,000_-*b*-PEO_6,500_). Cyclic voltammetry (CV) was used to determine the appropriate deposition potential (Fig. 3a). The presence of polymeric micelles has only a negligible influence on the positions of the cathodic peaks (Fig. S3), indicating that the micelles are electrochemically inert and primarily act as physical templates rather than redox-active species. Two cathodic peaks are observed: the peak at −0.14 V corresponds to the reduction of Ag^+^ to metallic Ag (Fig. S4a), whereas the peak at −0.44 V is assigned to the formation of Ag_2_Se.^51,52^ The corresponding cathodic peaks shift slightly toward more positive potentials, and their current densities increase in the second and subsequent scans, suggesting that an electrochemically active seed layer is formed during the first scan.^53^ In electrolytes containing polymer micelles, the cathodic current density is slightly reduced, likely because the micelles hinder the mass transport of Ag^+^ and Se(iv) species near the electrode surface. The sole reduction of Se(iv) species in the absence of Ag^+^ occurs only at a more negative potential than −0.7 V (Fig. S4b). The fact that Ag_2_Se formation takes place at −0.44 V, therefore, indicates that interactions between Ag^+^ and Se(iv) species facilitate the reduction of selenite and promote Ag_2_Se formation at significantly more positive potentials. In the micelle-free solution, no distinct oxidation peak associated with metallic Ag is observed in the reverse scan, which may arise from complete conversion of the Ag surface to Ag_2_Se and passivation by Se^0^ formed during the cathodic sweep.

**Fig. 3:**
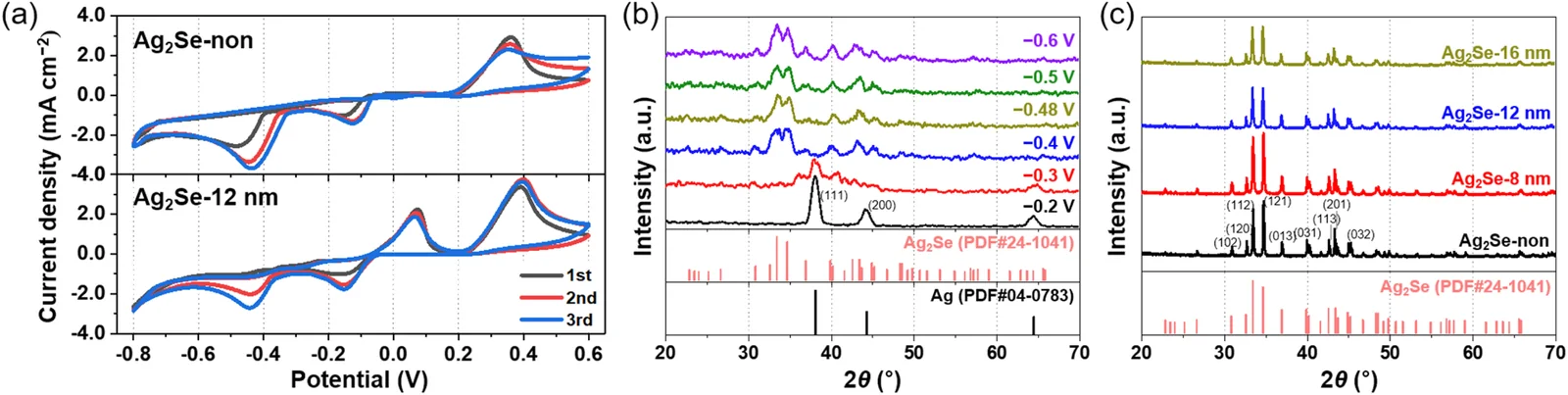
(a) CV curves of Ag_2_Se deposition electrolytes with and without PS_9,000_-*b*-PEO_3,500_, recorded at a scan rate of 20 mV s^−1^ between −0.8 and 0.6 V. An ITO substrate, a Pt wire, and a Hg/Hg_2_SO_4_ electrode were used as the working, counter, and reference electrodes, respectively. (b) XRD patterns of Ag_2_Se thin films electrodeposited at different reduction potentials. (c) PXRD patterns of Ag_2_Se particles electrodeposited at −0.48 V using different *block* copolymers and subsequently detached from the ITO substrates.

For film fabrication, constant potentials between −0.2 and −0.6 V were applied for 1000 s, and the resulting lattice phases were evaluated. At a given potential, the current–time profiles are similar with and without the soft template, differing only slightly in magnitude (Fig. S4c). As shown in Fig. 3b, X-ray diffraction (XRD) analysis reveals that films deposited at −0.2 V are composed exclusively of metallic Ag (PDF#04-0783),^54^ those deposited at −0.3 V contain a mixture of Ag and additional unidentified phases, and those obtained at potentials between −0.4 and −0.6 V consist solely of Ag_2_Se (PDF#24-1041), with no detectable Ag.^55,56^ This evolution corroborates the CV assignment that the first cathodic peak corresponds to Ag^+^ + e^−^ → Ag^0^ and the second peak to Ag_2_Se formation. Considering the pore morphology and Ag : Se ratio observed by scanning electron microscopy (SEM), discussed below, a deposition potential of −0.48 V was selected as optimal for preparing mesoporous Ag_2_Se.

Because lattice structure and grain size play critical roles in determining thermal conductivity, the electrodeposited films were further characterized by powder XRD (PXRD) and rietveld refinement. Ag_2_Se films grown on ITO glasses were delaminated by ultrasonication and thoroughly washed with 1.0 M HNO_3_ solution, water, and THF prior to analysis. Irrespective of the presence or type of polymer in the deposition bath, all diffraction patterns can be indexed to the orthorhombic β-Ag_2_Se phase with space group *P*2_1_2_1_2_1_ (Fig. 3c). Rietveld refinement of the PXRD data shows that the samples electrodeposited at −0.48 V are essentially single-phase β-Ag_2_Se,^57^ with only negligible amounts of secondary phases (Fig. S5). However, Ag_2_Se deposited at an excessively negative potential (−0.7 V) contains substantial secondary phases, including Se^0^ (Fig. S6).^58^ The refined crystallite size decreases from 167.6 nm for Ag_2_Se-non to 107.4, 102.1, and 89.0 nm for Ag_2_Se-8 nm, Ag_2_Se-12 nm, and Ag_2_Se-16 nm, respectively. Thus, while the polymer micelles do not alter the crystallographic phase of the deposited Ag_2_Se, they modestly suppress crystallite growth, indicating that the micelles act primarily as physical templates.

### Electron microscopy analysis of the pore architecture of Ag_2_Se

The pore architectures of mesoporous Ag_2_Se films were examined by scanning electron microscopy (SEM). To verify whether the pores are distributed throughout the films, cross-sectional SEM analysis was performed on samples deposited on fluorine-doped tin oxide (FTO) substrates, which provide cleaner fracture cross-sections than ITO. As a first step, the reduction potential required to obtain a well-defined pore structure together with an Ag : Se ratio close to 2 : 1 was screened between −0.2 and −0.6 V. A distinct porous morphology is observed only for films deposited between −0.4 and −0.48 V (Fig. S7). However, energy-dispersive X-ray spectroscopy (EDS) reveals that the film grown at −0.4 V contains 71 at% Ag, exceeding the 66 at% expected for stoichiometric Ag_2_Se (Fig. S8). Considering the deposition rate, pore morphology, and Ag : Se ratio, −0.48 V was therefore selected as the optimal deposition potential.

Because Ag_2_Se adheres poorly to ITO glass, applying a constant cathodic potential directly to the bare ITO substrate results in films with non-uniform coverage and locally aggregated regions (Fig. S9a and b). To improve nucleation and film continuity, a seed layer was first generated by CV, scanning the potential between −0.8 and 0.6 V starting from the open-circuit potential. The substrate was then gently wiped with a soft cloth to redistribute the nucleated Ag_2_Se seeds across the ITO surface, followed by potentiostatic deposition at −0.48 V. This procedure yields continuous films with a smooth, laterally homogeneous surface (Fig. S9c).

Top-view SEM images show that Ag_2_Se-non exhibits a relatively dense morphology with a moderately rough surface (Fig. 4a). Cross-sectional SEM (Fig. 4e) reveals occasional microcavities within the Ag_2_Se-non film, which are likely generated intrinsically during electrodeposition. In contrast, the mesoporous Ag_2_Se samples display a high density of uniformly sized pores across the entire film (Fig. 4b–d). The characteristic pore diameter increases systematically with the molecular weight of the polymer: the average pore diameters are 8 nm, 12 nm, and 16 nm for Ag_2_Se-8 nm, Ag_2_Se-12 nm, and Ag_2_Se-16 nm, respectively (Fig. S10). Small-angle X-ray scattering (SAXS) analysis indicates that the pores are distributed relatively uniformly. Ag_2_Se-non shows no distinct peak in the mesopore regime, whereas Ag_2_Se-8 nm, Ag_2_Se-12 nm, and Ag_2_Se-16 nm each exhibit a single broad maximum, consistent with a well-defined characteristic mesopore length scale but without long-range periodic ordering (Fig. S11). The film thicknesses are 300 nm for Ag_2_Se-non, 350 nm for Ag_2_Se-8 nm, 400 nm for Ag_2_Se-12 nm, and 450 nm for Ag_2_Se-16 nm (Fig. 4e–h), despite identical deposition times and applied potentials. This trend is consistent with an increased effective film volume associated with the incorporation of internal mesopores.

**Fig. 4:**
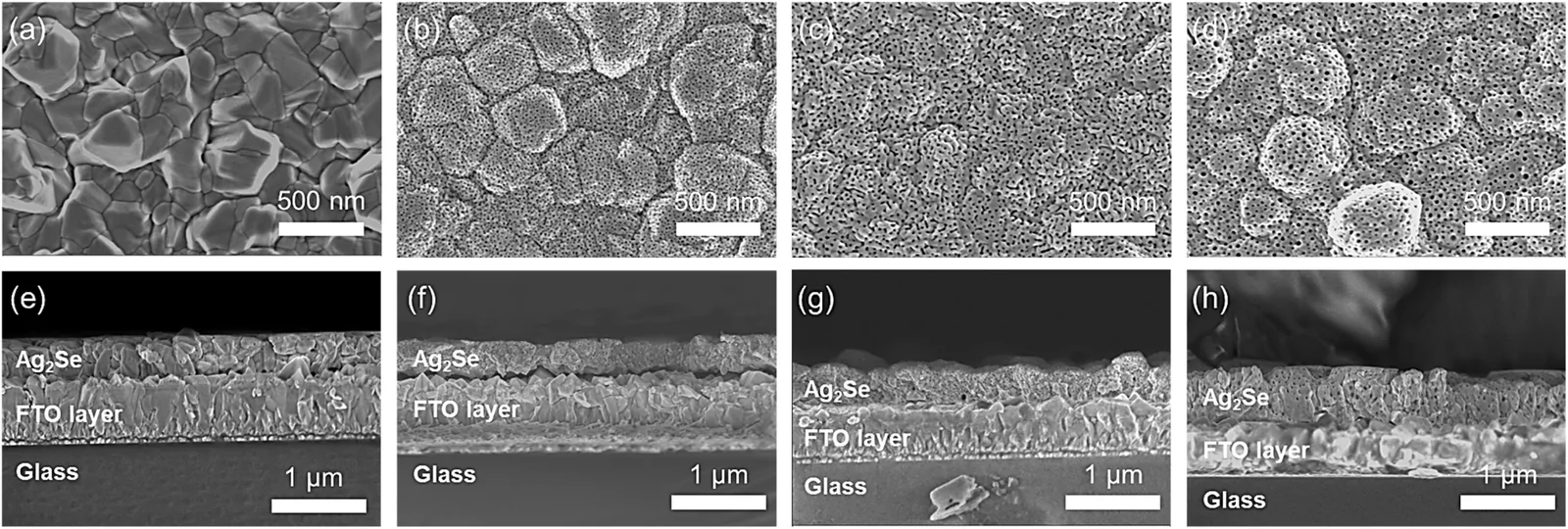
(a–d) Top-view and (e–h) cross-sectional SEM images of Ag_2_Se films: (a and e) Ag_2_Se-non, (b and f) Ag_2_Se-8 nm, (c and g) Ag_2_Se-12 nm, and (d and h) Ag_2_Se-16 nm.

Ag_2_Se films electrodeposited on ITO glasses were detached by ultrasonication, thoroughly washed, and collected as fine particles with lateral dimensions of several tens of micrometers (Fig. S12). SEM images of these particles show that the mesoporous architecture is essentially preserved upon detachment, and surface contaminants present in the as-deposited films are more effectively removed, yielding a more clearly resolved pore structure. Fourier-transform infrared spectroscopy (FTIR) analysis further supports the effective removal of residual PS-*b*-PEO polymer after washing (Fig. S13).

Transmission electron microscopy (TEM) analysis confirms the presence of internal pores within the individual particles and reveals lattice fringes with a *d*-spacing of 0.27 nm (Fig. 5a–c), which corresponds to the (112) plane of orthorhombic β-Ag_2_Se in the XRD pattern. Scanning TEM-EDS (STEM-EDS) elemental mapping further shows that Ag and Se are homogeneously distributed throughout the particles (Fig. 5d), with no evidence of phase segregation or agglomeration. Taken together, these observations demonstrate that the Ag_2_Se particles detached from ITO substrates retain both the original mesoporous architecture and the intrinsic β-Ag_2_Se lattice structure.

**Fig. 5:**
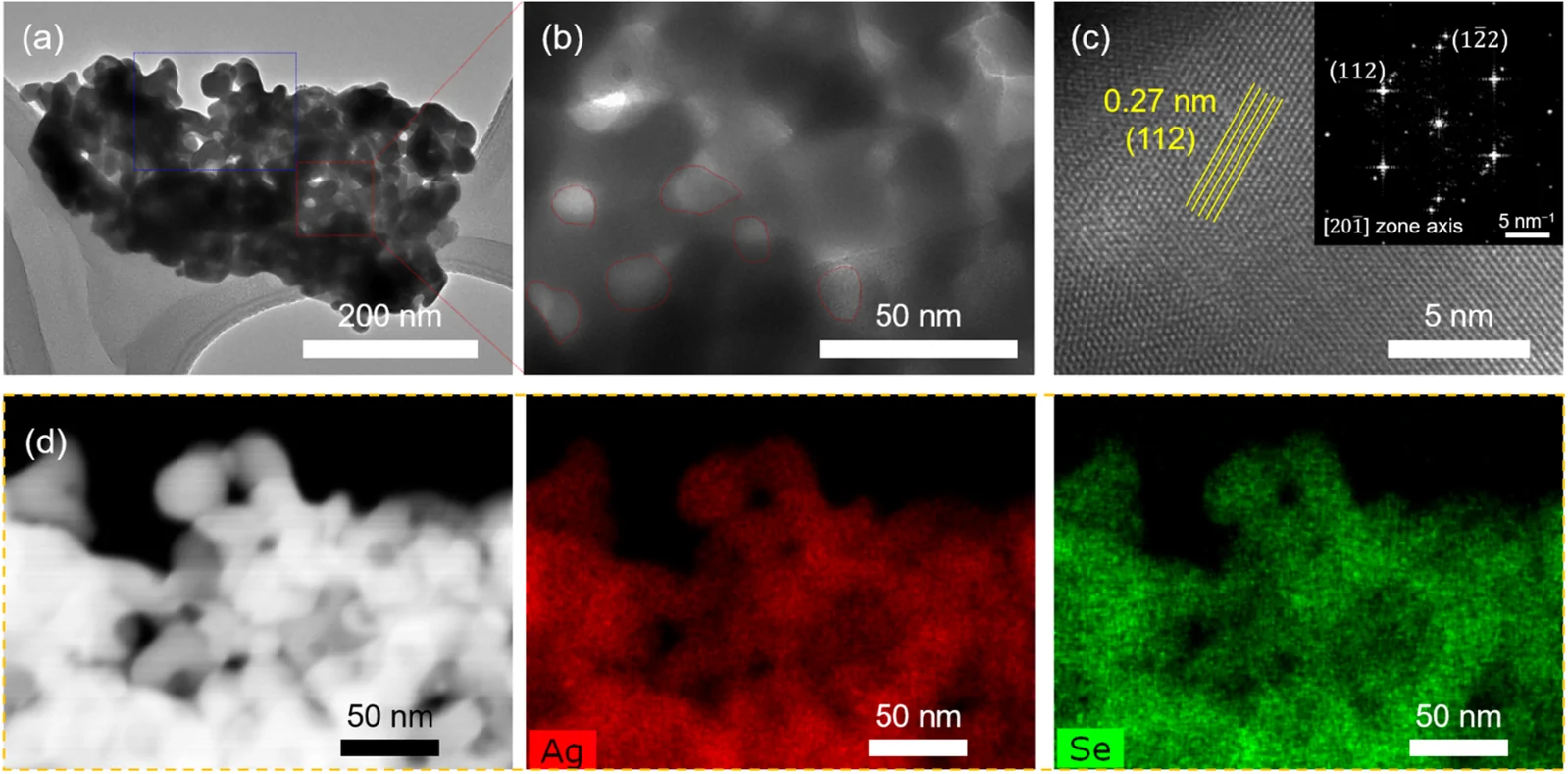
(a) TEM image of mesoporous Ag_2_Se-12. (b) Enlarged TEM image of the red-boxed region in (a), showing the mesoporous morphology. The red line outlines the pore. (c) High-resolution TEM image and corresponding local fast Fourier transform (FFT) pattern of Ag_2_Se. The measured lattice spacing of 0.27 nm is assigned to the (112) plane of orthorhombic Ag_2_Se, and the FFT pattern is consistent with the [20̄1] zone axis. (d) STEM-EDS analysis of the blue-boxed region in (a), including the corresponding STEM image and elemental maps of Ag and Se.

### Thermal conductivity and thermoelectric performance of mesoporous Ag_2_Se

Prior to the experimental study, the influence of mesopores embedded in the Ag_2_Se lattice on thermal conductivity was evaluated using *ab initio*-based molecular dynamics (MD) simulations (Fig. S14a).^59,60^ Due to computational constraints, the supercell size and pore diameters were limited to approximately 110 Å and several tens of angstroms, respectively (Fig. S14b), yet even within this restricted parameter space the introduction of pores consistently reduced the calculated thermal conductivity. Theoretical calculations show a clear monotonic decrease of *κ*_L_ with increasing temperature for a nonporous system (Fig. S14c). This tendency is aligned with enhanced anharmonic phonon–phonon scattering at higher temperatures, which shortens the phonon mean free path and suppresses thermal conduction. Notably, introducing porosity produces an additional, strong reduction of *κ*_L_ (Fig. S14d). Mechanistically, mesopores act as additional phonon scattering centers through boundary scattering and local strain fields near the pore surface, which further reduce phonon lifetimes and mean free paths.^36,61^ Moreover, enlarging the pore increases the internal surface area and decreases the effective heat-carrying cross section, both of which amplify the resistance to heat flow.

The thermoelectric performance of mesoporous Ag_2_Se was evaluated using pellets cold-pressed from fine particles detached from ITO substrates (Fig. S15). To avoid any thermal history near the β → α phase transition of Ag_2_Se (∼407 K),^62^ which could potentially perturb the pore architecture, the powders were pressed at room temperature. The resulting pellet densities are 6.79, 6.80, 6.71, and 6.73 g cm^−3^ for Ag_2_Se-non, Ag_2_Se-8 nm, Ag_2_Se-12 nm, and Ag_2_Se-16 nm, respectively—substantially lower than the crystalline density of β-Ag_2_Se (∼8.2 g cm^−3^), reflecting the presence of internal porosity and residual interparticle voids in the pressed pellets. Nevertheless, the pellets were mechanically robust enough for reliable thermoelectric measurements.

As shown in Fig. 6a, Ag_2_Se-non exhibits a *σ* of 1565 S cm^−1^ at 298 K, which gradually decreases with increasing temperature. In contrast, the mesoporous samples show an overall increase in *σ* with temperature, suggesting that the temperature dependence of charge transport is modified by the mesoporous microstructure and associated grain-boundary characteristics. The smaller refined crystallite sizes of the mesoporous Ag_2_Se samples, approximately 90–110 nm compared with 167.6 nm for Ag_2_Se-non, suggest an increased grain-boundary density. This increased density of interfacial barriers may have promoted thermally activated carrier transport, contributing to the semiconducting-like increase in *σ* with temperature.^63,64^ Additionally, the mesoporous samples do not exhibit a simple monotonic decrease in *σ* despite their higher porosity, indicating that the electrical transport is not governed solely by the reduced conductive volume. Ag_2_Se-12 nm displays a lower *σ* of 1420 S cm^−1^ at 298 K than Ag_2_Se-non, which can be partly attributed to the reduced effective electronic percolation pathway caused by its porous framework. In contrast, Ag_2_Se-8 nm and Ag_2_Se-16 nm exhibit slightly higher *σ* than Ag_2_Se-non. The different temperature dependence of *σ* likely reflects the combined effects of carrier concentration, mobility, grain-boundary characteristics, and conductive connectivity in the porous pellets. Hall measurements help rationalize this trend (Fig. S16): although the mesoporous samples show lower Hall carrier concentrations (*n*_H_) than Ag_2_Se-non, they exhibit markedly higher Hall mobilities (*µ*_H_). These results suggest that the decrease in *n*_H_ induced by the mesoporous structure is compensated for, or even overcompensated, by enhanced *µ*_H_ in selected samples. Therefore, the mesoporous framework appears to influence charge transport through a balance between reduced conductive volume, modified *n*_H_, and mobility-governing microstructural factors, including conductive connectivity, crystallographic arrangement, grain-boundary characteristics, and local defect states.^40,65^ Such differences may originate from interactions between polymer micelles and precursor ions during synthesis, which could alter the local stoichiometry and defect environment of the resulting porous Ag_2_Se framework.

**Fig. 6:**
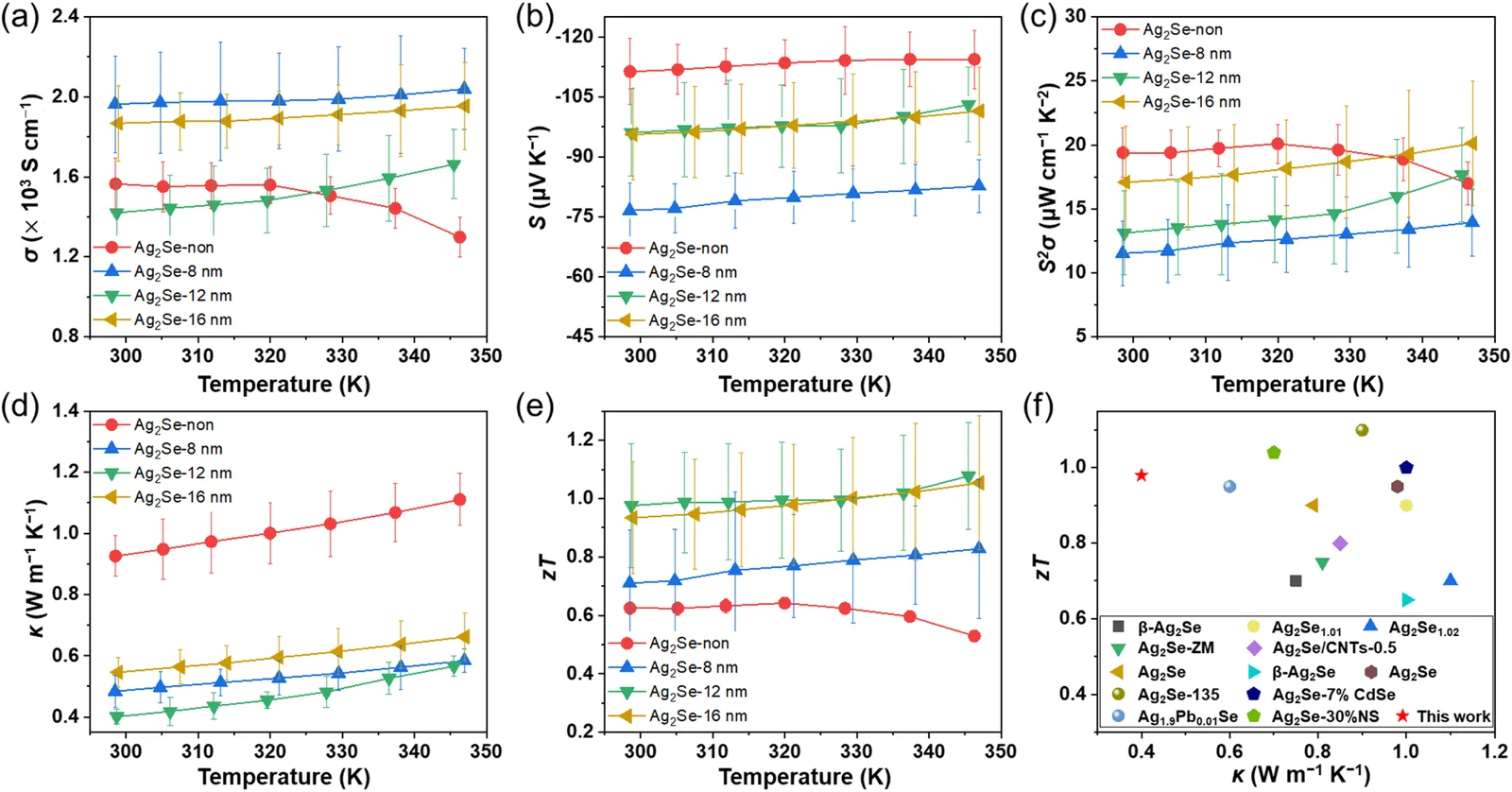
(a–e) Temperature-dependent (a) electrical conductivity *σ*, (b) Seebeck coefficient *S*, (c) power factor *S*^2^*σ*, (d) thermal conductivity *κ*, and (e) *zT* of the Ag_2_Se samples. (f) Comparison of near-room-temperature *zT* and thermal conductivity values of bulk-type Ag_2_Se-based thermoelectrics reported in the literature with Ag_2_Se-12 nm.^43,61,70,71,77–84^

For Ag_2_Se-non, temperature-dependent Hall measurements further indicate that the decrease in *σ* above ∼330 K is mainly governed by mobility degradation rather than by a decrease in *n*_H_ (Fig. S16b). The decrease in *σ* despite the increase in *n*_H_ indicates that enhanced carrier scattering dominates the high-temperature transport behavior. This reduction in *µ*_H_ can be attributed to intensified lattice vibrations and stronger electron–phonon scattering at elevated temperatures, which are likely facilitated by the soft, anharmonic lattice nature of β-Ag_2_Se.^66,67^

Fig. 6b summarizes the *S* measured from 298 to 347 K. All samples exhibit negative *S* values, confirming electron-majority (n-type) transport. Unlike the trend in *σ*, the magnitude of *S* increases slightly with temperature for all samples. Ag_2_Se-non shows the largest magnitude, changing from −111.3 µV K^−1^ at 298 K to −114.4 µV K^−1^ at 347 K. In contrast, the mesoporous samples display slightly smaller *S* values (*e.g.*, Ag_2_Se-12 nm: −96.0 µV K^−1^ at 298 K and −103.0 µV K^−1^ at 347 K). Although the mesoporous samples exhibit lower *n*_H_ than Ag_2_Se-non, their smaller |*S*| values suggest that the thermoelectric transport of these pellets is not governed solely by *n*_H_.^68,69^ This result implies that the porous framework may modify additional transport descriptors beyond the apparent *n*_H_, such as the scattering mechanism, conductivity-weighted transport pathways, or local defect states. Fig. 6c shows the temperature-dependent PFs. For Ag_2_Se-non, the PF exhibits a shallow maximum within the measured range, reflecting the competing temperature dependences of *σ* and *S*. By comparison, the mesoporous samples show a monotonic increase in PF up to 347 K. At 298 K, Ag_2_Se-12 nm delivers a PF of 13.11 µW cm^−1^ K^−2^, whereas Ag_2_Se-16 nm reaches 17.08 µW cm^−1^ K^−2^, primarily due to its higher *σ* while maintaining comparable *S*.

To determine the *κ* of the pelletized samples, thermal diffusivity (*α*) was measured by the laser-flash method (LFA), and *κ* was calculated using *κ* = *αρC*_p_, where *ρ* is the pellet density, and *C*_p_ is the specific heat capacity.^70^ Since the synthesized Ag_2_Se is polycrystalline and the pellets were pressed without intentional texturing, the measured *κ* values are taken to represent the bulk-averaged thermal transport in this temperature range. Ag_2_Se-non shows *κ* of 0.93 W m^−1^ K^−1^ at 298 K (Fig. 6d), in line with typical room-temperature values reported for bulk β-Ag_2_Se.^71^ Remarkably, all mesoporous samples exhibit lower *κ* at 298 K, with Ag_2_Se-12 nm reaching 0.40 W m^−1^ K^−1^ (a 57% reduction). This reduction likely reflects the combined influence of several structural factors, including insulating voids, additional phonon-boundary scattering associated with the mesoporous structure and grain boundaries, and the reduced crystallite size induced by micelle-templated growth.^72^ Because these structural features are intrinsically coupled in the present samples, their individual contributions cannot be quantitatively separated. Nevertheless, from a kinetic viewpoint, *κ*_L_ scales with the effective phonon mean free path (MFP), and nanoscale pore interfaces can provide additional boundaries for phonon scattering, particularly for phonon modes whose intrinsic MFP is comparable to or larger than the characteristic structural length scale. In β-Ag_2_Se—characterized by a soft, anharmonic lattice^67^—the combined effects of mesostructural boundary scattering and reduced crystallite size are therefore expected to suppress phonon propagation while only moderately perturbing electronic transport.

The *κ*_e_ − *κ*_L_ separation based on the conventional Wiedemann–Franz (WF) relation (*κ*_e_ = *LσT*, where *L* is the Lorenz number) should be interpreted cautiously for the present mesoporous Ag_2_Se pellets. The Lorenz number is not universal in thermoelectric semiconductors and can deviate from the Sommerfeld value depending on carrier statistics, band structure, and scattering mechanisms.^73–75^ The Kim–Snyder estimate of the Lorenz number assumes a single parabolic band dominated by acoustic phonon scattering, conditions that are unlikely to be satisfied in mesoporous β-Ag_2_Se, where multi-band contributions and modified scattering at pore walls and grain boundaries are expected. Combined with the known sensitivity of the Lorenz number to nonstoichiometric point defects in β-Ag_2_Se,^76^ the apparent *κ*_e_ > *κ* observed in the WF treatment (Fig. S17) indicates that the conventional decomposition is not applicable here. We therefore report *κ* as the primary transport descriptor and avoid an explicit *κ*_e_ − *κ*_L_ separation, while noting that the systematic *κ* reduction with mesoporosity (Fig. 6d) provides direct evidence of suppressed heat transport independent of this decomposition.

To verify whether the mesoporous structure was retained after compression and thermoelectric measurements (Fig. S18), the measured pellets were crushed and examined by SEM. The SEM images show that numerous mesopores remain after cold pressing, although the average pore size is modestly reduced. Partial pore deformation and localized pore coalescence are also observed in highly compressed regions.

Fig. 6e summarizes the temperature-dependent *zT* from 298 to 347 K. Ag_2_Se-non exhibits a *zT* of 0.63 at 298 K, which gradually decreases to 0.53 at 347 K, primarily reflecting the reduction in PF. Notably, Ag_2_Se-12 nm delivers the highest *zT* among the mesoporous samples, reaching 0.98 at 298 K (a 56% enhancement relative to Ag_2_Se-non) and increasing further to 1.08 at 347 K. This improvement arises from a markedly suppressed *κ* that more than compensates for the moderate PF reduction in the mesoporous samples. The resulting *zT* values, driven by unusually low *κ*, compare favorably with recent Ag_2_Se-based thermoelectrics reported near room temperature (Fig. 6f).^43,61,70,71,77–84^ Collectively, these results demonstrate that mesoscale pore engineering can suppress heat transport to an extent that outweighs the accompanying reduction in electronic performance, thereby enhancing the overall thermoelectric performance of Ag_2_Se.

## Conclusions

In summary, we report the first realization of mesoporous Ag_2_Se prepared *via* a *block*-copolymer-directed soft-templated electrodeposition route, establishing a solution-processable route to introduce well-defined mesoporosity into a high-performance thermoelectric semiconductor. ELS provides direct evidence that PS-*b*-PEO micelles interact with both Ag^+^ and Se(iv) species in the precursor solution, supporting a mechanism in which ion-micelle interactions localize the precursors around the micelles and ultimately enable the micelles to be incorporated as structure-directing entities during Ag_2_Se growth. By controlling the reduction potential, we simultaneously tune the Ag : Se stoichiometry and the resulting pore architecture, yielding orthorhombic β-Ag_2_Se with uniformly distributed mesopores. The resulting mesoporous Ag_2_Se exhibits a pronounced suppression of heat transport: the thermal conductivity is reduced to 0.40 W m^−1^ K^−1^ at room temperature. As a result, Ag_2_Se-12 nm achieves a *zT* of 0.98 at 298 K, representing a substantial enhancement over the nonporous counterpart. These results differ from conventional Ag_2_Se thermoelectric studies, in which improvements in *zT* typically rely on compositional optimization to maximize the PF, yet are ultimately limited by the intrinsic trade-off between the Seebeck coefficient and electrical conductivity that arises from their shared dependence on carrier concentration and scattering. Instead, our work demonstrates that controlled mesoporosity provides an effective microstructural strategy for suppressing thermal conductivity in an intrinsically strong thermoelectric host.

## Experimental section

### Chemicals

All chemicals and solvents were used as received without further purification. Silver nitrate (AgNO_3_, ≥98%), selenium dioxide (SeO_2_, ≥99.9%), 70% nitric acid (HNO_3_), *N*,*N*-dimethylformamide (DMF), and tetrahydrofuran (THF) were purchased from Sigma-Aldrich (USA). *Block* copolymers (BCPs) with different molecular weights, PS_5,000_-*b*-PEO_2,500_, PS_9,000_-*b*-PEO_3,500_, and PS_19,000_-*b*-PEO_6,500_ (where the numbers indicate molecular weights), were obtained from Polymer Source Inc. All solutions were prepared with deionized water (DIW) treated with a Millipore water purification system.

### Characterization

Zeta potential measurements were carried out by ELS using an ELSZ-2000 instrument (Otsuka Electronics Co., Ltd, Japan). ELS measurements were repeated three times for each sample, and the reported values represent the averages of the three independent measurements. The morphology and composition of the samples were analyzed using field emission scanning electron microscopy (FE-SEM, JSM-7100F, JEOL Ltd) equipped with energy-dispersive X-ray spectroscopy (EDS), operated at accelerating voltages of 2.0 kV for imaging and 15.0 kV for EDS. Pore sizes were determined from multiple SEM images of the Ag_2_Se-8 nm, Ag_2_Se-12 nm, and Ag_2_Se-16 nm samples using ImageJ software, with at least 100 pores measured for each sample to ensure statistical reliability. X-ray diffraction (XRD) patterns were collected on a SmartLab diffractometer (Rigaku Corp.) using Cu Kα radiation to determine the crystal structure. Phase identification was carried out using QualX2,^85^ and structural parameters were refined by Rietveld analysis with the Profex program.^86^ Lattice-structure information for Ag_2_Se and Se^0^ was taken from the Crystallography Open Database (https://www.crystallography.net/cod/). The microstructures were further examined by transmission electron microscopy (TEM) and scanning TEM (STEM) using a Tecnai G2 F20 instrument (FEI Co.) operated at an accelerating voltage of 80 kV. The Seebeck coefficient and electrical resistivity were measured simultaneously under an Ar atmosphere using the temperature differential and four-probe methods (ZEM-3(M8), ULVAC), respectively, over the 298–347 K temperature range. The measurements were repeated three times, and the reported values represent the averages of the three measurements; the corresponding error bars indicate the measurement variation. The cold-pressed pellets had a diameter of approximately 7 mm and a thickness of approximately 1 mm. Pellets with the same geometry were used for measurements of electrical transport properties and thermal conductivity. The carrier concentration and Hall mobility of the samples were measured using a Hall effect measurement system (Ecopia, HMS-5300) in the Van der Pauw configuration over the temperature range of 298–347 K, with each measurement repeated three times. The magnetic field was 0.575 T, and the applied current was set between 100 µA and 20 mA. The in-plane thermal diffusivity (*D*) and specific heat (*C*_p_) of the pellets were measured using the laser-flash technique (LFA-457, NETZSCH) with reference to Pyroceram standard calibration and differential scanning calorimeter (DSC 200 F3 Maia, NETZSCH) in the temperature range from 298 K to 347 K. The measurement was conducted using a dedicated in-plane holder and analyzed with an anisotropic model. The in-plane thermal conductivity (measured along the same direction as the electrical conductivity and Seebeck coefficient) was calculated using *κ* = *ρDC*_p_, where *ρ* represents the pellet density.

### Preparation of electrolyte for electrodeposition of Ag_2_Se film

5 mg of PS-*b*-PEO was dissolved in 1.5 ml of DMF and 0.5 ml of ethanol under stirring. After complete dissolution, the synthesis bath was prepared by sequentially adding 0.5 ml of 80 mM AgNO_3_, 2.0 ml of 3 M HNO_3_, and 0.5 ml of 80 mM SeO_2_. To synthesize Ag_2_Se films with different pore sizes, PS_5,000_-*b*-PEO_2,500_, PS_9,000_-*b*-PEO_3,500_, and PS_19,000_-*b*-PEO_6,500_ were used, yielding Ag_2_Se-8 nm, Ag_2_Se-12 nm, and Ag_2_Se-16 nm, respectively.

### Electrochemical synthesis of Ag_2_Se films

Electrochemical experiments, including CV and electrodeposition, were performed in a three-electrode configuration using a CHI 760E electrochemical workstation (CHI Instruments, USA). An indium tin oxide (ITO) or fluorine-doped tin oxide (FTO) glass, a platinum (Pt) wire, and a Hg/Hg_2_SO_4_/saturated K_2_SO_4_ electrode (RE-5B) served as the working, counter, and reference electrodes, respectively. ITO glass with a sheet resistance of 8–12 Ω sq^−1^ and FTO glass with a sheet resistance of ∼7 Ω sq^−1^ were purchased from Sigma-Aldrich, and the Pt wire and RE-5B were obtained from BAS Inc. (Japan). Before use, the ITO glass was cleaned *via* plasma treatment (Filgen, Japan).

For Ag_2_Se deposition, a seed layer was first generated by CV, sweeping the potential from the open-circuit potential to −0.8 V and then to 0.6 V. The electrode surface was subsequently gently wiped with a soft cloth to redistribute the Ag_2_Se seeds. Subsequently, Ag_2_Se films were grown potentiostatically by applying a constant potential of −0.48 V for 1000 s. After electrodeposition, the films were rinsed with THF at 40 °C to remove the PS-*b*-PEO template and then dried in a vacuum oven for 4 h.

Ag_2_Se particles were obtained by detaching the deposited films from the ITO substrates *via* mild ultrasonication in a 1.0 M HNO_3_ solution. The collected particles were washed three times each with water and THF to remove residual impurities and polymer species and then dried at 60 °C for 12 h.

## Author contributions

Z. Yang: investigation, formal analysis, visualization, writing – original draft. M. Han: conceptualization, methodology, data curation, writing – original draft, writing – review & editing. J. Kim: formal analysis. B. Kim: formal analysis. H. N. Nam: methodology, software, formal analysis. Q. M. Phung: software, formal analysis. Y. J. Lee: formal analysis. N. C.-R. Chen: formal analysis. X. Liu: formal analysis. Y. Guo: formal analysis. Y. Zhao: formal analysis. Y. Kang: formal analysis. K. C.-W. Wu: formal analysis. Y. Asakura: formal analysis. Y. Yamauchi: conceptualization, supervision, project administration, funding acquisition, writing – review & editing.

## Conflicts of interest

There are no conflicts to declare.

## Supplementary Material

SC-OLF-D6SC05923F-s001

## Data Availability

The data supporting this article have been included as part of the supplementary information (SI). Supplementary information: photographs demonstrating the Tyndall effect, CV curves of Ag_2_Se synthesis electrolytes, Rietveld refinement of the obtained mesoporous Ag_2_Se particles, SEM images and SEM-EDS analyses of mesoporous Ag_2_Se films prepared under various conditions, statistically analyzed pore-size distributions, SAXS patterns of mesoporous Ag_2_Se, FTIR spectra, theoretical calculation methods and results, and Hall measurement results (SI Fig. S1–S18); solution compositions for zeta-potential measurements (Table S1) and a comparison of the room-temperature *zT* values of pelletized Ag_2_Se (Table S2). See DOI: https://doi.org/10.1039/d6sc05923f.
