# Supplementary material for: Mesoporous Ag2Se as a model n-type thermoelectric: soft-templated synthesis and thermal transport engineering
Source: Chem Sci. 2026 Oct 13. Online ahead of print. doi: 10.1039/d6sc05923f (PMC13659298; doi:10.1039/d6sc05923f)
Supplement: Supplementary file 1 [file SC-OLF-D6SC05923F-s001.pdf]

Supplementary Information (SI) for Chemical Science.
This journal is © The Royal Society of Chemistry

Supporting Information

Mesoporous Ag_2Se as a Model n-Type Thermoelectric: Soft-Templated Synthesis and Thermal Transport Engineering

Zihao Yang,[a,‡] *Minsu Han,*[a,b,‡*] *Jihyun Kim,*[c] *Ho Ngoc Nam,*[a] *Quan Manh Phung,*[d,e] *Young Jun Lee,*[f] *Norman C.-R. Chen,*[a,g,h] *Xiangyang Liu,*[a] *Yuqi Guo,*[a] *Yingji Zhao,*[a] *Yunqing Kang,*[a,b] *Kevin C.-W. Wu,*[g,h,i,j] *Yusuke Asakura,*[a] *Byeonggwan Kim,*[c] *Yusuke Yamauchi*[a,b,k]*

[a]Department of Materials Process Engineering, Graduate School of Engineering, Nagoya University, Nagoya 464-8603, Japan

[b]Australian Institute for Bioengineering and Nanotechnology (AIBN), The University of Queensland, Brisbane, QLD 4072, Australia

[c]Department of Chemical Engineering and Applied Chemistry, Chungnam National University, 99 Daehak-ro, Yuseong-gu, Daejeon 34134, Republic of Korea

[d]Department of Chemistry, Graduate School of Science, Nagoya University, Nagoya 464-8602, Japan.

[e]Institute of Transformative Bio-Molecules (WPI-ITbM), Nagoya University, Nagoya 464-8601, Japan.

[f]Carbon Composite Materials Research Center, Korea Institute of Science and Technology (KIST), 92 Chudong-ro, Bongdong-eup, Wanju-gun, Jeollabuk-do 55324, Republic of Korea

[g]Molecular Science and Technology Program, Taiwan International Graduate Program, Academia Sinica, Taipei, 10617, Taiwan

[h]International Graduate Program of Molecular Science and Technology (NTU-MST), National Taiwan University, Taipei, 10617, Taiwan

[i]Department of Chemical Engineering, National Taiwan University, Taipei, 10617, Taiwan

[j]Center of Atomic Initiative for New Materials (AI-MAT), National Taiwan University, Taipei, 10617, Taiwan

[k]Department of Chemical and Biomolecular Engineering, Yonsei University, 50 Yonsei-ro, Seodaemun-gu, Seoul 03722, Republic of Korea

‡These authors contributed equally.

*Corresponding authors: Minsu Han (minsu.han@nagoya-u.jp); Yusuke Yamauchi (y.yamauchi@uq.edu.au)

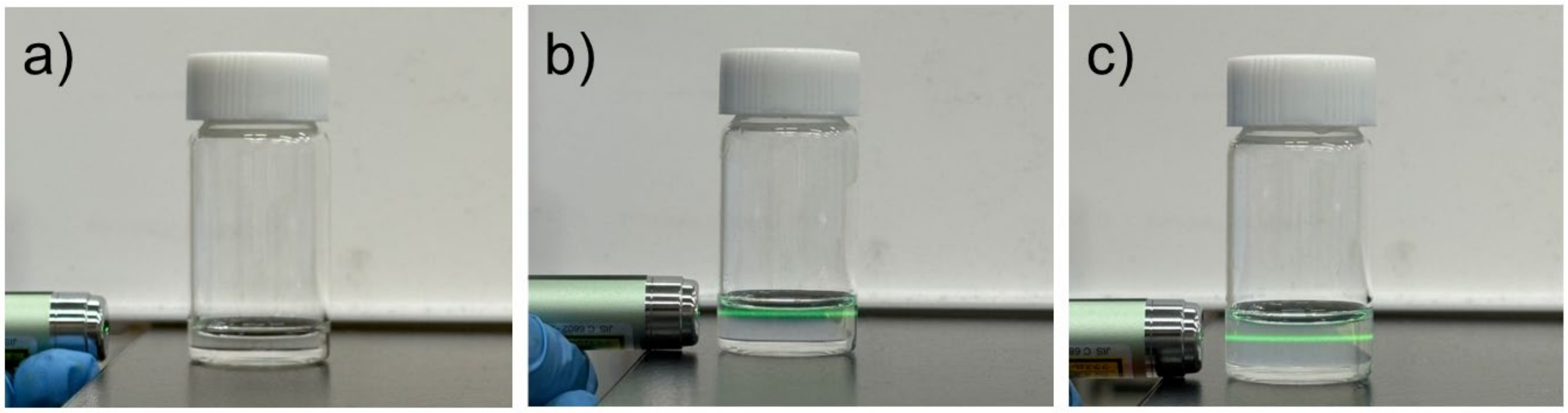

Figure S1. Photographs demonstrating the Tyndall effect in solutions with different compositions: (a) PS-b-PEO + DMF + ethanol, (b) PS-b-PEO + DMF + ethanol + HNO_3, and (c) PS-b-PEO + DMF + ethanol + HNO_3 + $AgNO_3$ + SeO_2.

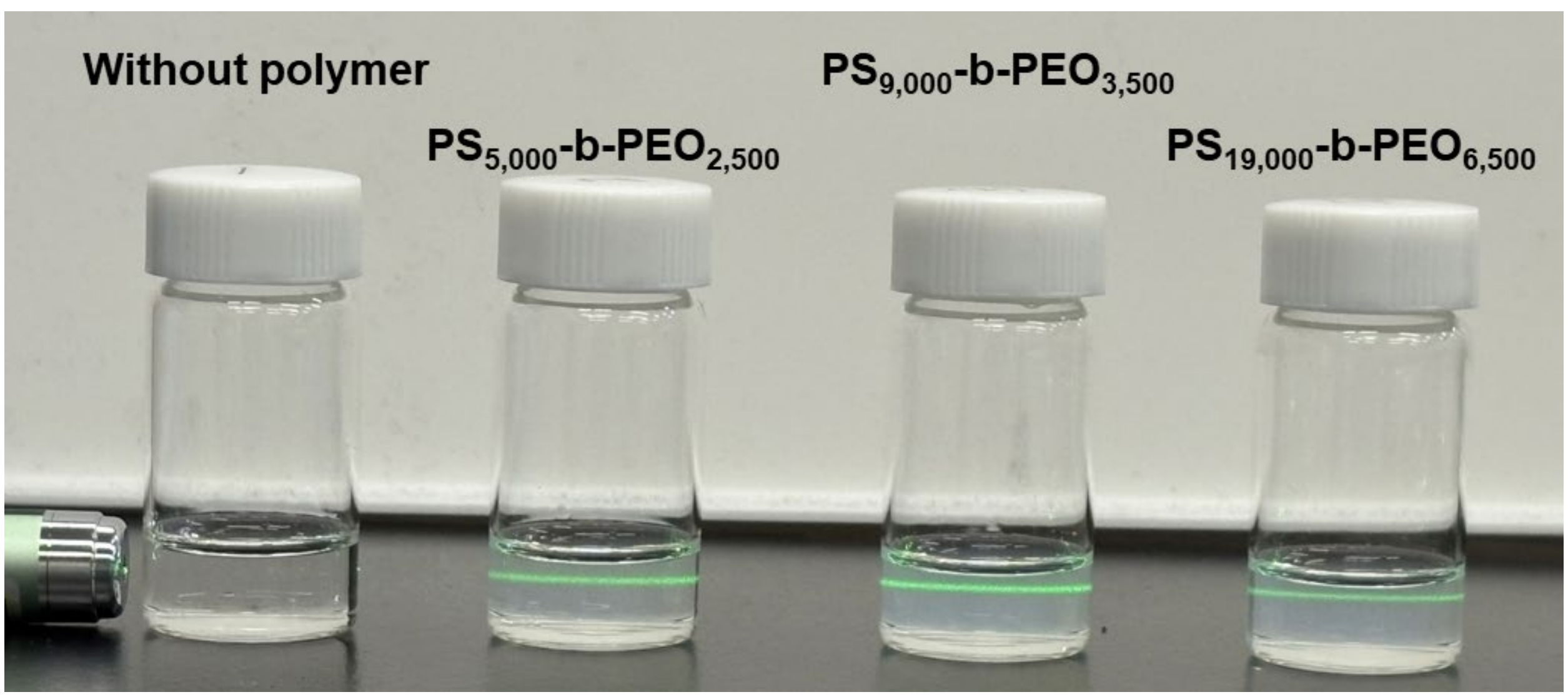

Figure S2. Comparison of the Tyndall effect in mixed solutions with and without polymer, and with different polymer molecular weights.

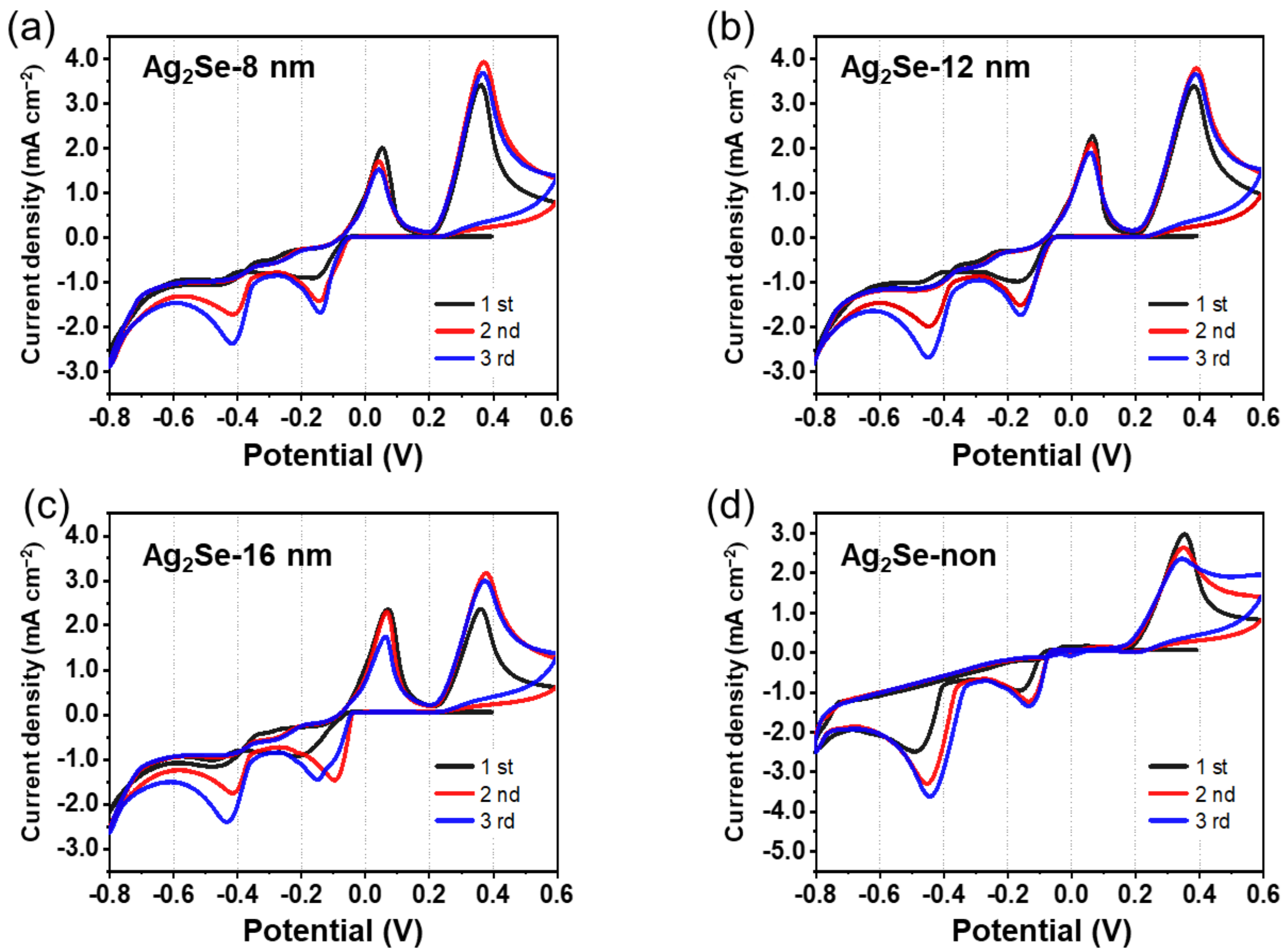

Figure S3. Cyclic voltammograms (CVs) of electrolytes for Ag_2Se synthesis containing different block copolymers, recorded at a scan rate of 20 mV s^{-1} from −0.8 to 0.6 V: (a) $PS_{5,000}$-b-$PEO_{2,500}$ (Ag_2Se-8 nm), (b) $PS_{9,000}$-b-$PEO_{3,500}$ (Ag_2Se-12 nm), (c) $PS_{19,000}$-b-$PEO_{6,500}$ (Ag_2Se-16 nm), and (d) without polymer (Ag_2Se-non). A Hg/Hg_2SO_4 reference electrode was employed to preserve Ag^+ in the electrolyte.

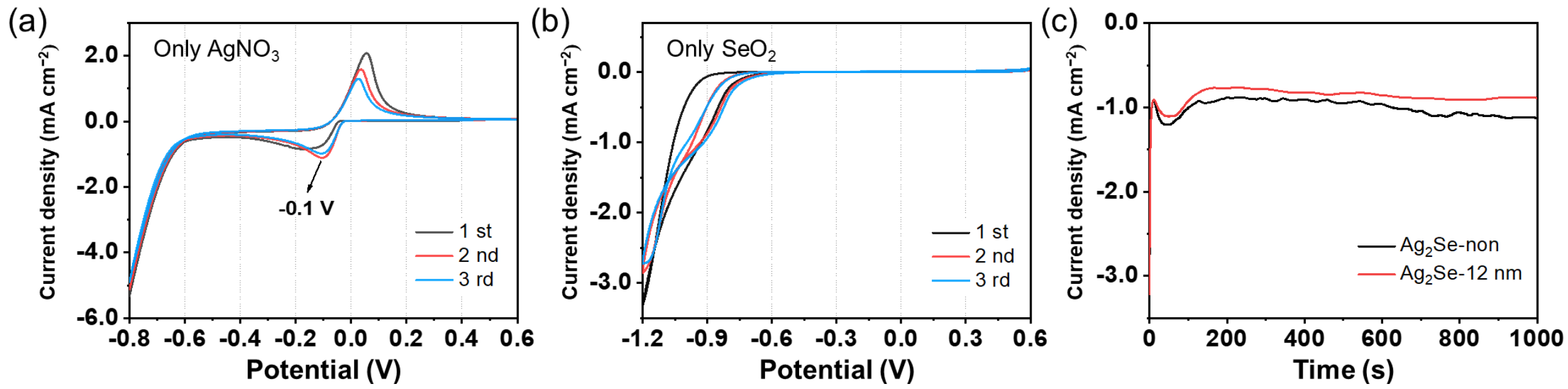

Figure S4. (a,b) CVs of electrolytes for Ag_2Se synthesis recorded in the absence of (a) SeO_2 and (b) $AgNO_3$, respectively. The scan rate was 20 mV s^{-1}, and $PS_{9,000}$-b-$PEO_{3,500}$ was used as a soft template. (c) Amperometric *i*–*t* curve recorded during the electrodeposition of Ag_2Se at −0.48 V.

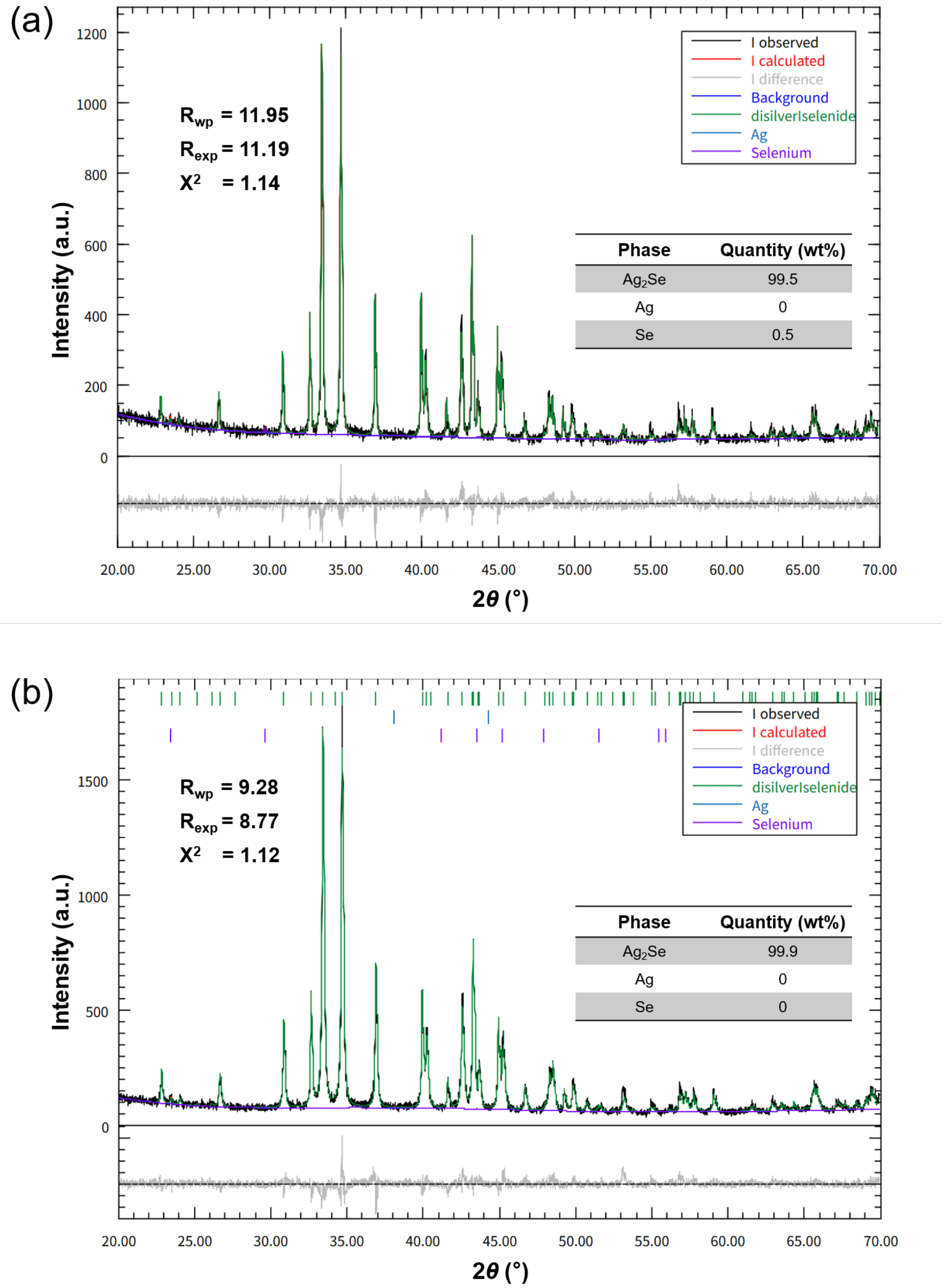
(a)
I observed
I calculated
I difference
Background
disilverIselenide
Ag
Selenium
R_{wp} = 11.95
R_{exp} = 11.19
X^2 = 1.14
Phase | Quantity (wt%)
Ag_2Se | 99.5
Ag | 0
Se | 0.5
Intensity (a.u.)
2θ (°)
(b)
I observed
I calculated
I difference
Background
disilverIselenide
Ag
Selenium
R_{wp} = 9.28
R_{exp} = 8.77
X^2 = 1.12
Phase | Quantity (wt%)
Ag_2Se | 99.9
Ag | 0
Se | 0
Intensity (a.u.)
2θ (°)

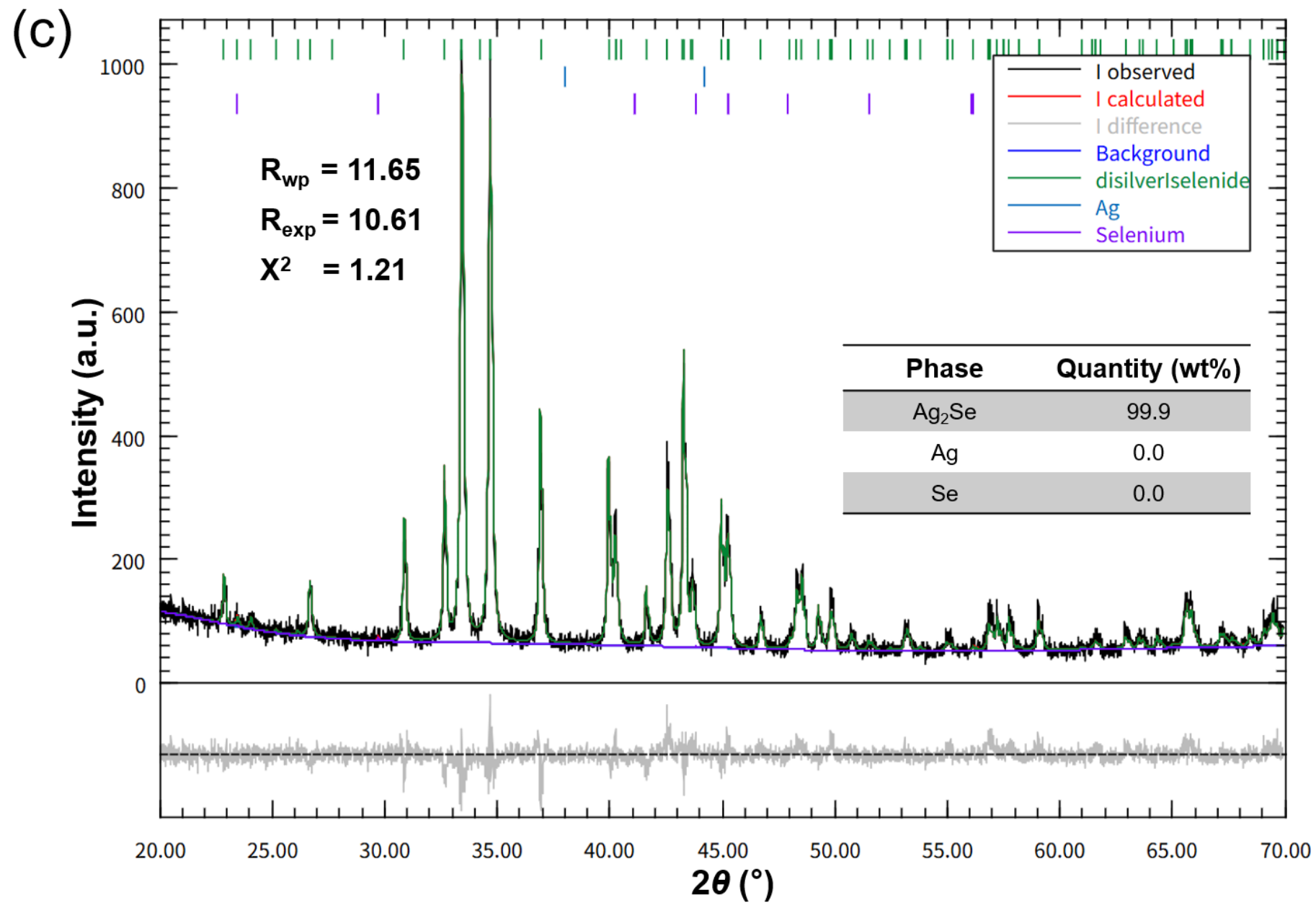

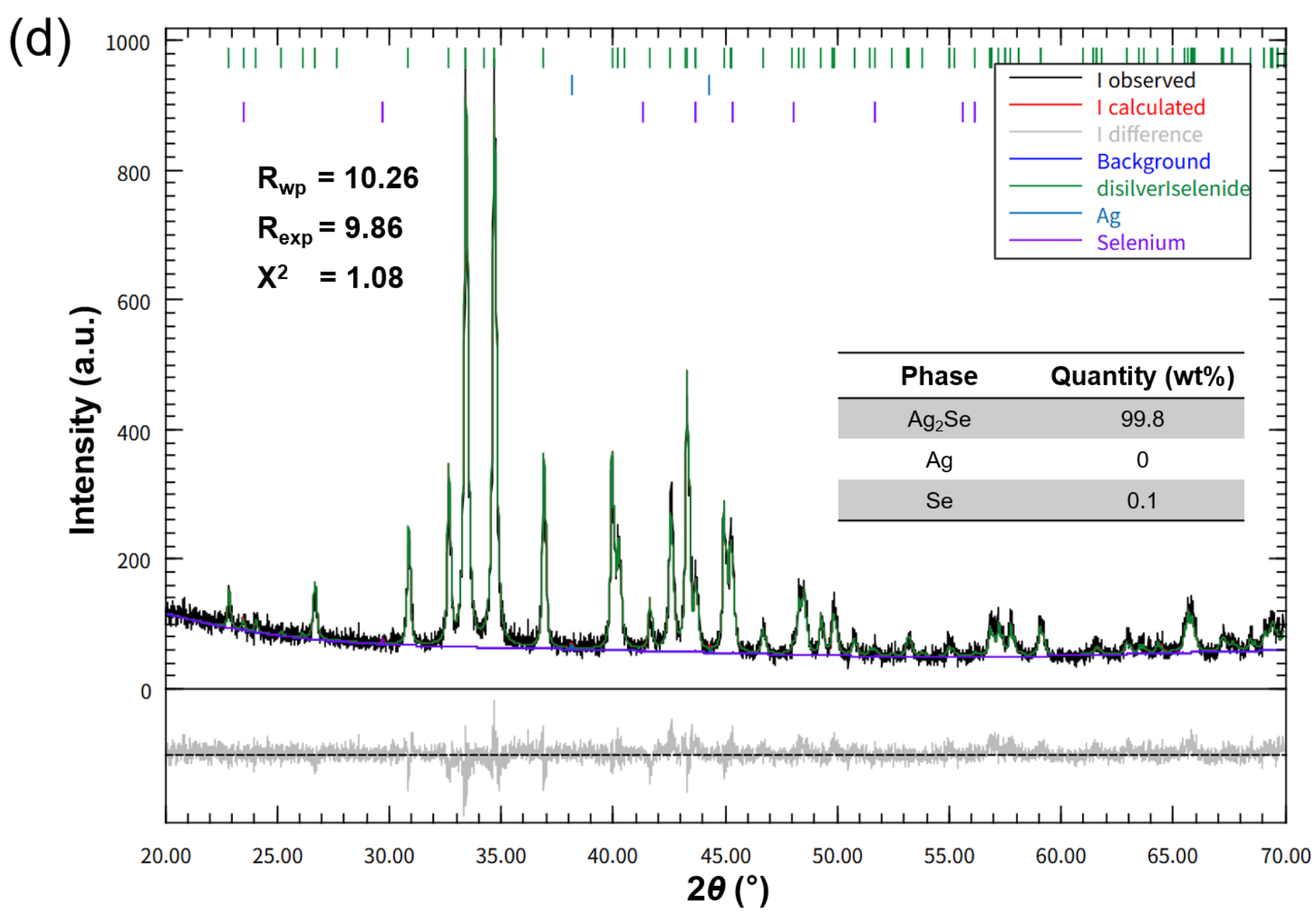

Figure S5. Rietveld refinement of the powder X-ray diffraction (PXRD) patterns of (a) Ag_2Se-non, (b) Ag_2Se-8 nm, (c) Ag_2Se-12 nm, and (d) Ag_2Se-16 nm to confirm the phase composition. Orthorhombic β-Ag_2Se (COD 2230972) was used as the structural model.

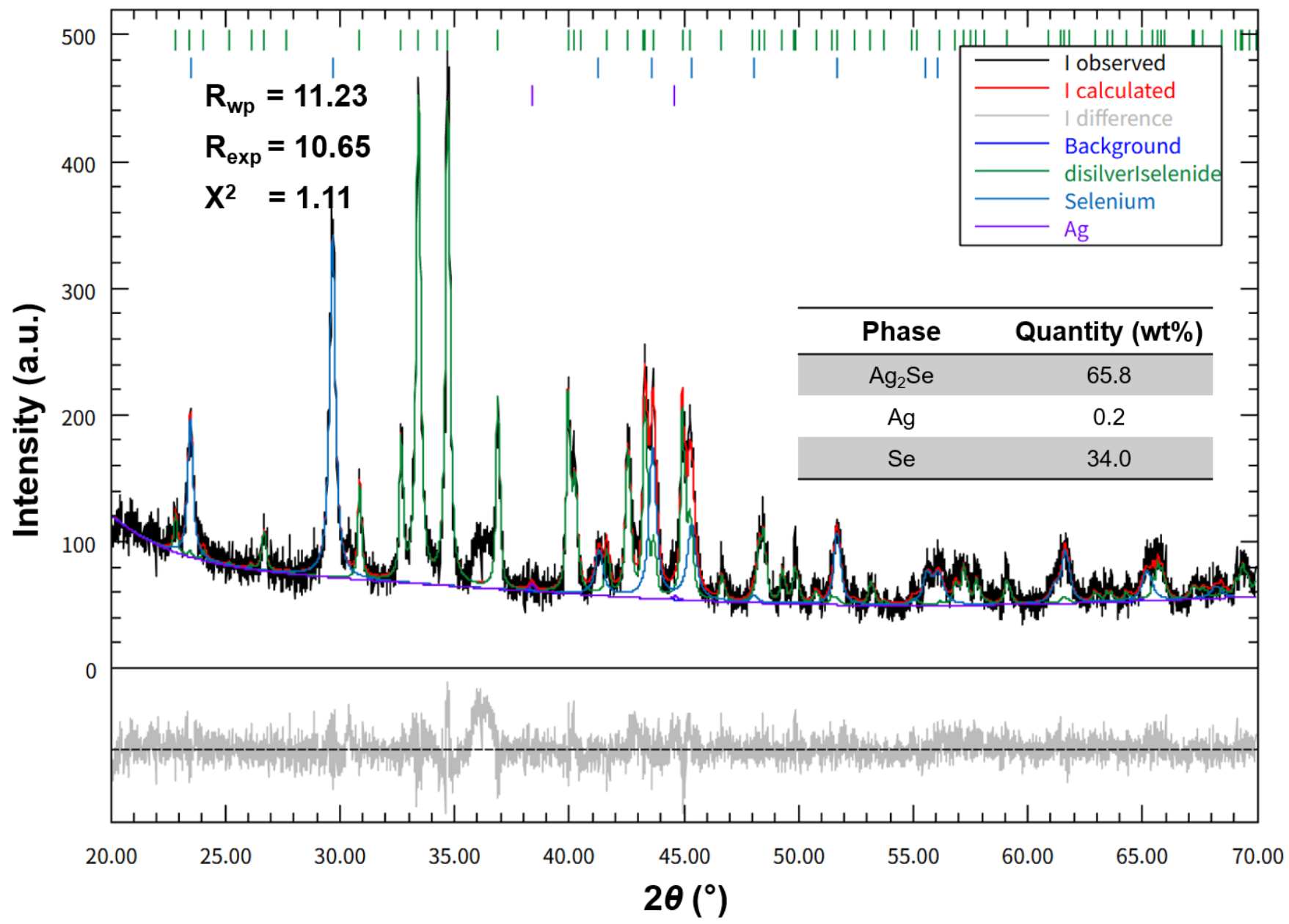

Figure S6. Rietveld refinement of the PXRD pattern of Ag_2Se-12 nm electrodeposited at −0.7 V to confirm the phase composition. Orthorhombic β-Ag_2Se (COD 2230972) and trigonal Se (COD 9011648) were used as the structural models.

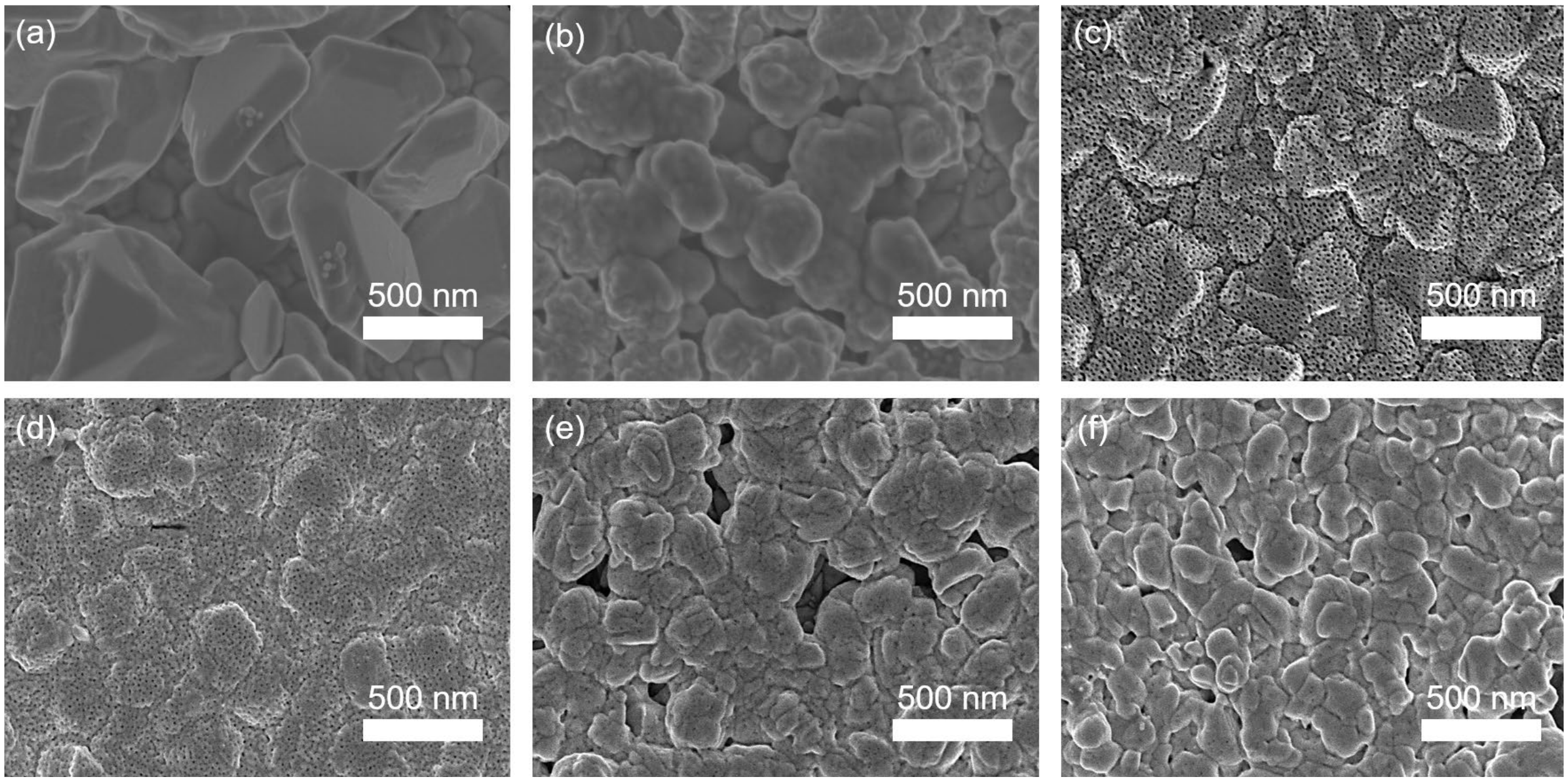

Figure S7. Top-view scanning electron microscope (SEM) images of Ag_2Se thin films electrodeposited at different reduction potentials for 1000 s: (a) −0.2 V, (b) −0.3 V, (c) −0.4 V, (d) −0.48 V, (e) −0.5 V, and (f) −0.6 V.

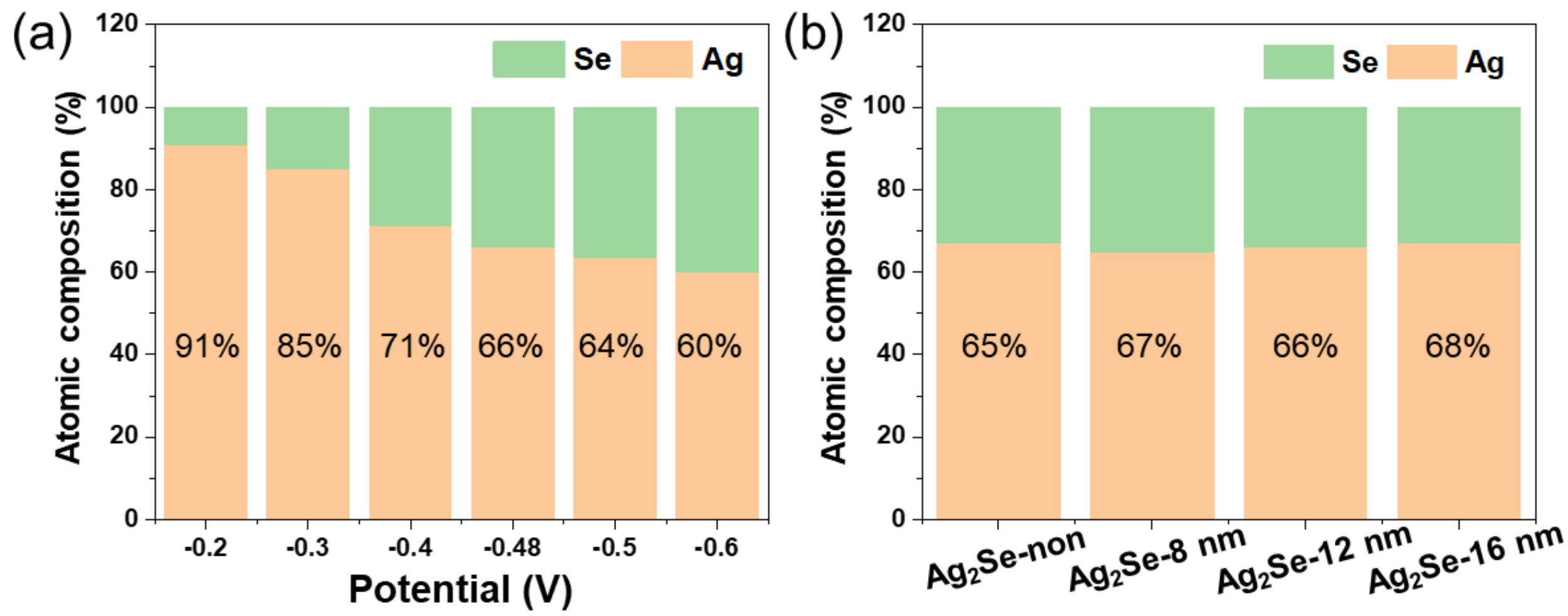

Figure S8. (a) SEM–energy-dispersive X-ray spectroscopy (SEM-EDS) analysis of Ag_2Se thin films electrodeposited at different reduction potentials. (b) SEM-EDS analysis of Ag2Se films prepared at −0.48 V using different block copolymers.

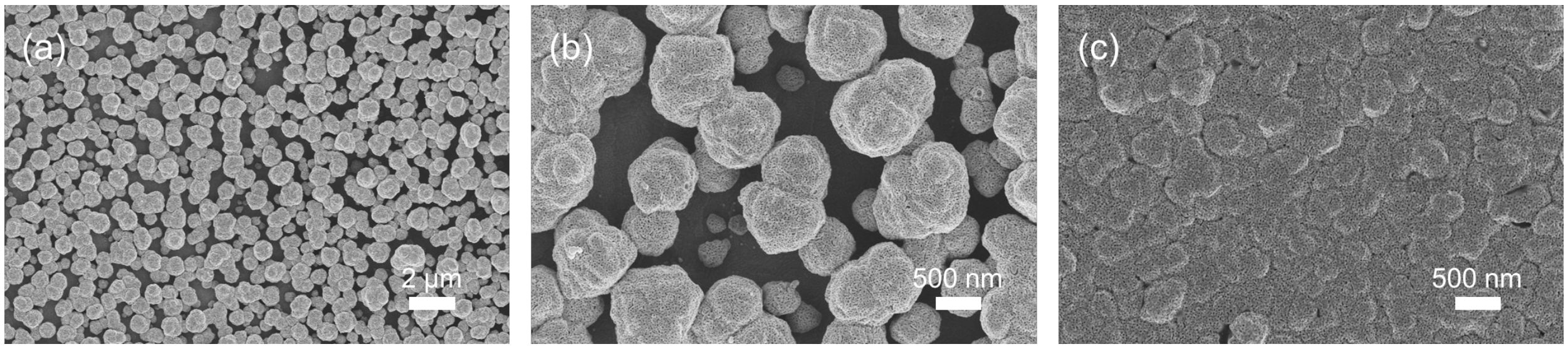

Figure S9. (a,b) SEM images of Ag_2Se films electrodeposited on ITO substrates by directly applying −0.48 V for 1000 s. (c) SEM image of an Ag_2Se film electrodeposited at −0.48 V for 1000 s after forming a seed layer by CV.

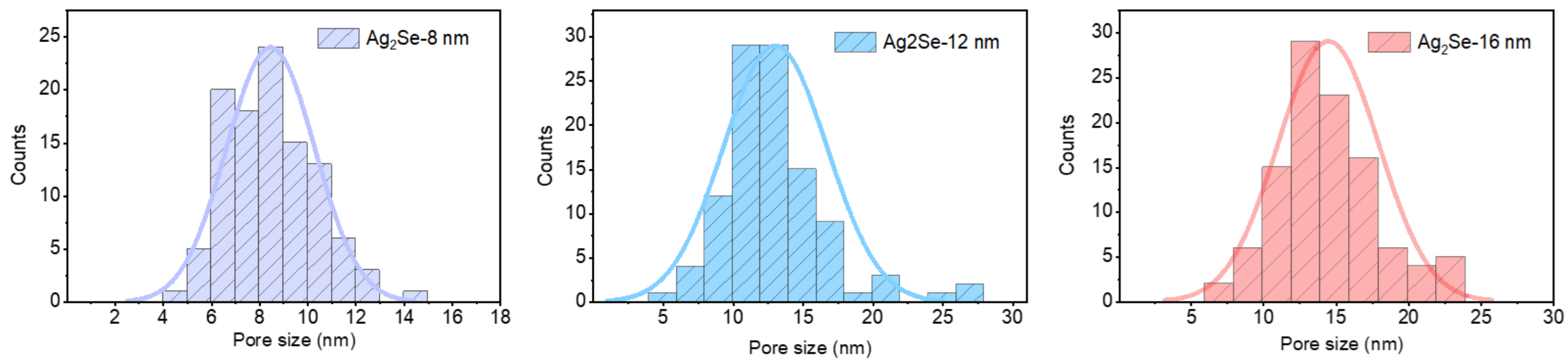

Figure S10. Pore-size distribution profiles of mesoporous Ag_2Se films synthesized using different block copolymers. All samples exhibit narrow, well-defined pore-size distributions.

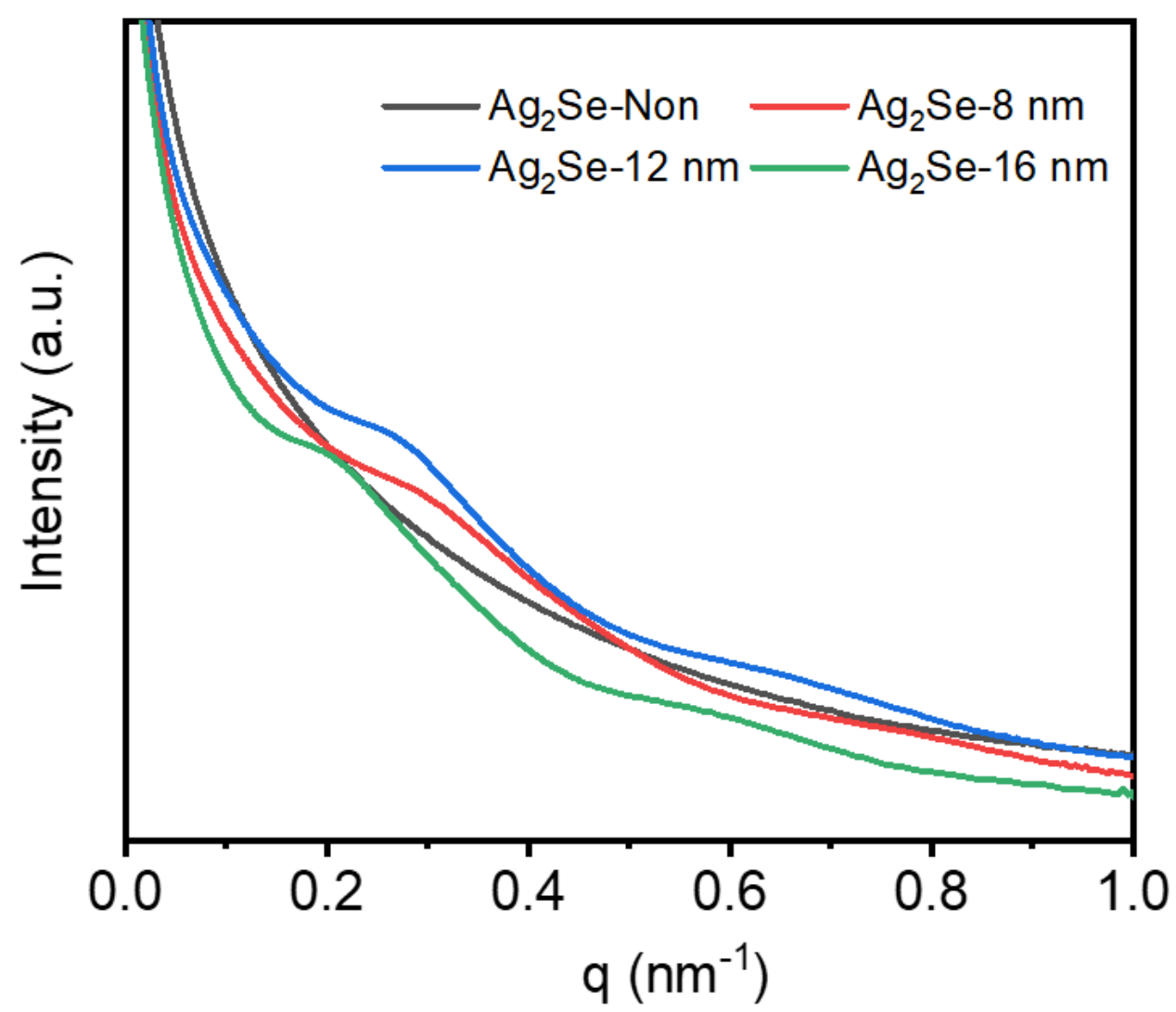

Figure S11. Small-angle X-ray scattering (SAXS) patterns of mesoporous Ag_2Se samples with different pore architectures.

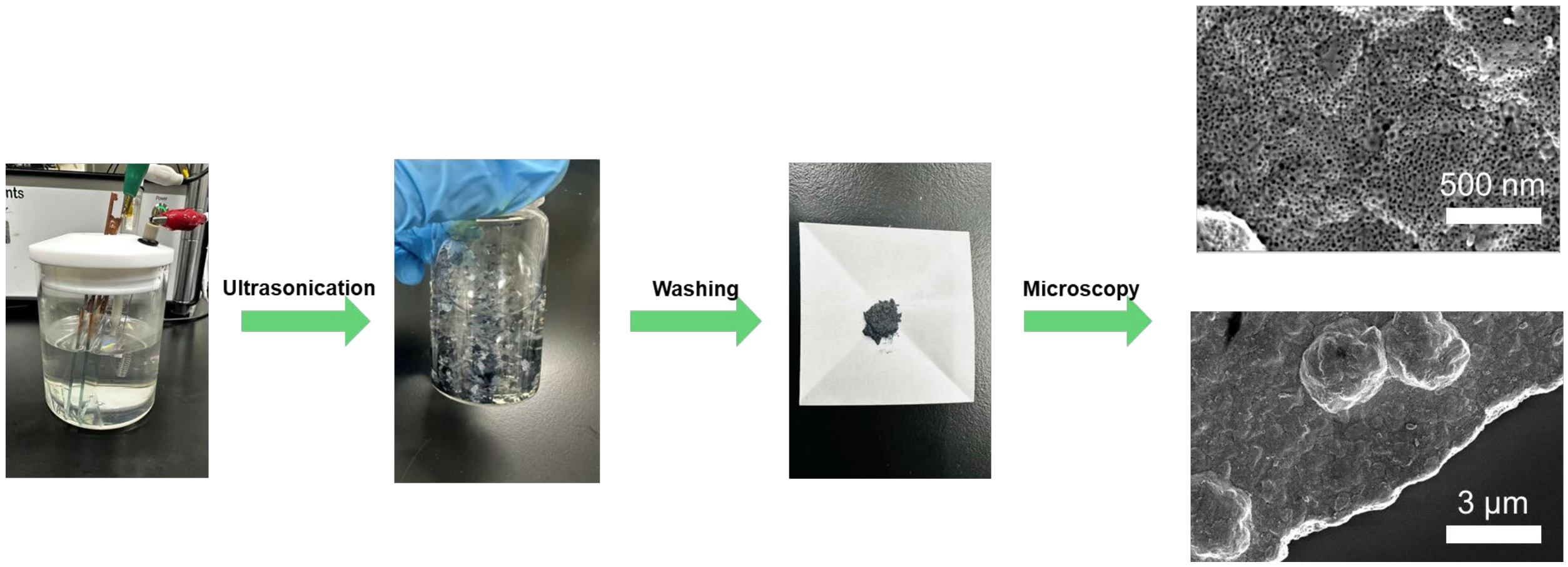

Figure S12. Photographic illustration of the procedure used to obtain freestanding Ag_2Se particles by delaminating electrodeposited Ag_2Se films from ITO glasses, and SEM images of the resulting Ag_2Se particles.

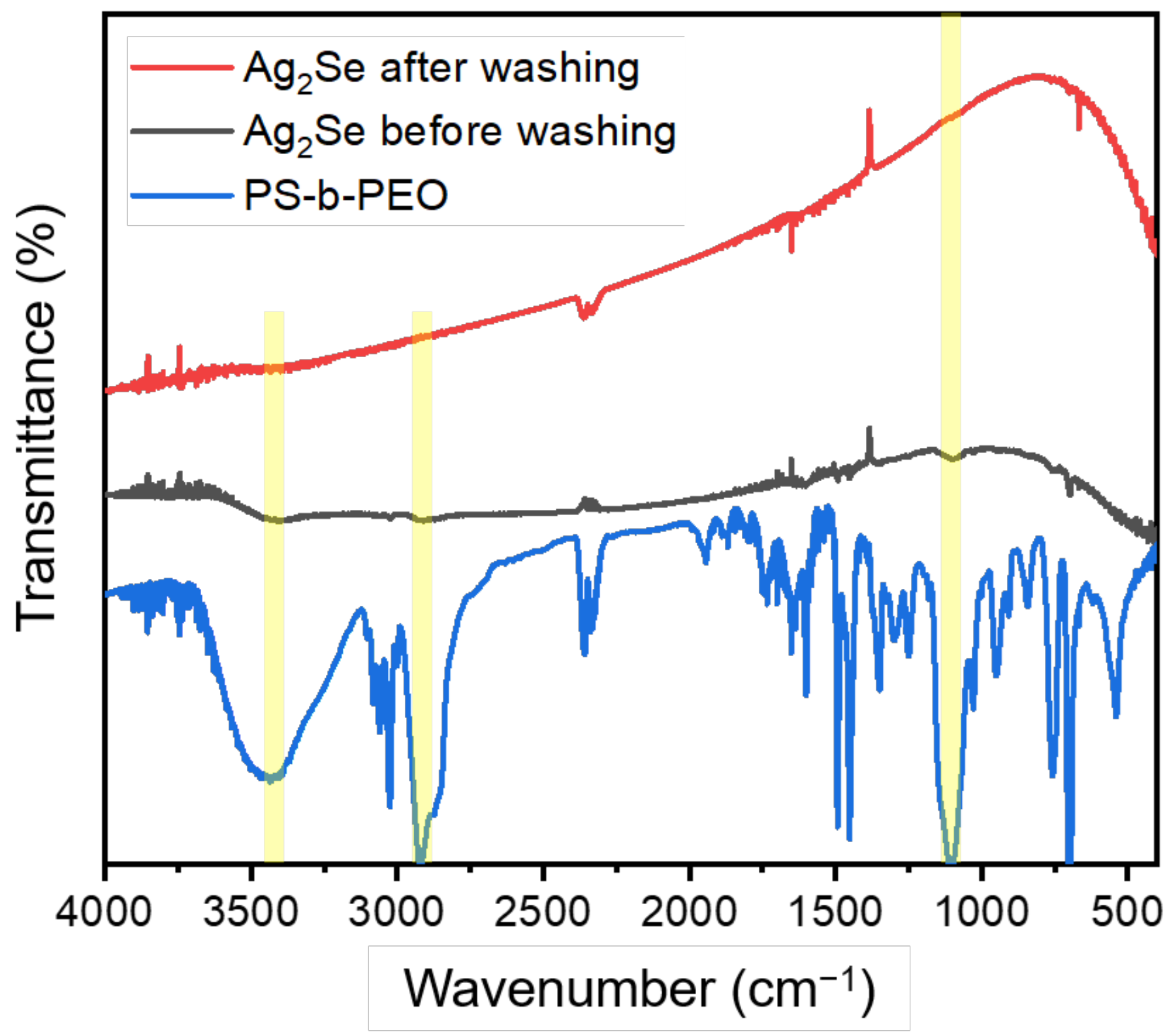

Figure S13. FTIR spectra of PS-b-PEO and Ag_2Se particles before and after washing. The marked suppression of the characteristic PS-b-PEO absorption bands after washing supports the effective removal of residual polymer.

Theoretical calculation methods

To investigate lattice thermal conductivity of Ag_2Se, *ab initio* molecular dynamics (AIMD) simulations were performed to generate reference data for constructing a machine-learning interatomic potential. Specifically, density functional theory (DFT) calculations were first carried out using the Vienna *Ab initio* Simulation Package (VASP).[1-3] The PBE approximation was employed together with Grimme's D3 dispersion correction with Becke-Johnson damping (PBE-D3(BJ)).[4, 5] The plane-wave kinetic energy cutoff was set to 300 eV, approximately 1.2 times the highest ENMAX among the PAW pseudopotentials used (249.8 eV for Ag and 211.5 eV for Se). The electronic self-consistent-field convergence criterion (EDIFF) was set to 10^{-4} eV. This cutoff value is consistent with the cutoff employed in a previous AIMD-based training-set generation study on the related silver chalcogenide Ag_2S.[6] A set of 10 representative systems was considered to ensure transferability of the potential, including bulk Ag_2Se in the orthorhombic phase, low-index surfaces ((001), (010), and (100)), an $Ag_{18}Se_{10}$ cluster, systems containing Ag and Se vacancies, and three porous Ag_2Se models with different pore sizes (see Figure S14). Subsequently, AIMD simulations were performed on these systems at 400 K, rather than at the target NEMD temperature of 300 K, in order to sample a broader range of structural distortions.[7] The simulations were performed using the atomic simulation environment (ASE) package,[8] employing a Langevin thermostat with a friction coefficient of 0.01 fs^{-1} and a time step of 3 fs. The simulation time was 30 ps (10000 steps), and atomic positions were saved every 100 MD steps. Reference energies and atomic forces from these AIMD trajectories were used for training the machine-learning potential using the DeepMD package.[9, 10] Finally, classical MD simulations were performed with this trained potential in LAMMPS[11, 12]. The lattice thermal conductivity was computed using nonequilibrium molecular dynamics (NEMD) simulations. [13, 14]

Four Ag_2Se model systems were constructed to investigate the effect of porosity on lattice thermal conductivity. All systems share similar simulation cell dimensions, with box lengths of L_x ~ 110 Å, L_y ~ L_z ~ 35 Å. Heat transport was studied along the *x*-direction. System 1 corresponds to a fully dense, nonporous Ag_2Se reference structure. Systems 2–4 are porous models containing a single cylindrical pore whose axis is aligned along the *y*-direction, i.e., perpendicular to the heat-transport direction. The three porous systems differ only in pore size, with average diameters of 16 Å (system 2), 20 Å (system 3), and 28 Å (system 4), respectively.

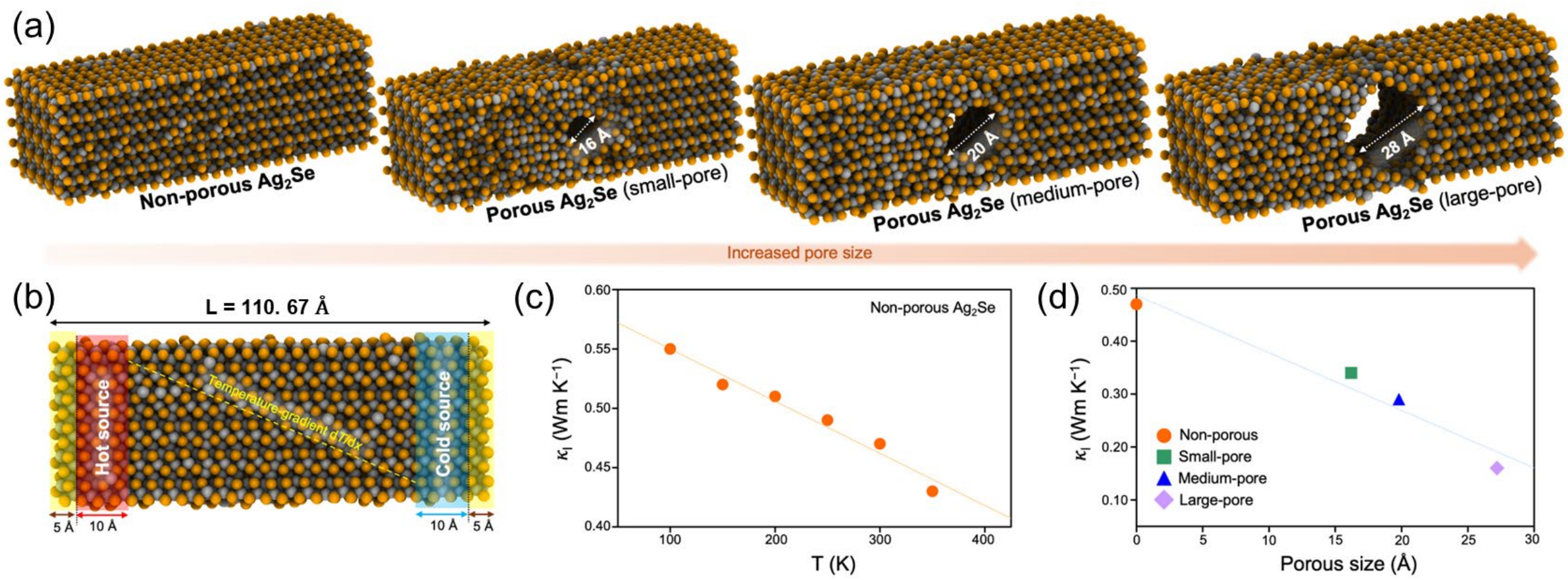

Figure S14. (a) Nonporous and three porous model systems used for heat-transport simulation. The average diameter for each porous model is in Å. (b) Setup for NEMD simulations: two thin slabs (5 Å thickness) at the boundaries were frozen and are indicated by yellow rectangles. The thermal reservoirs, each with a width of 10 Å, are highlighted in red (hot source) and blue (cold source). Color code: Ag (gray) and Se (orange). (c) The calculated lattice thermal conductivity of nonporous Ag_2Se as a function of temperature. (d) Effect of pore size/porosity on the lattice thermal conductivity of Ag_2Se at 300 K.

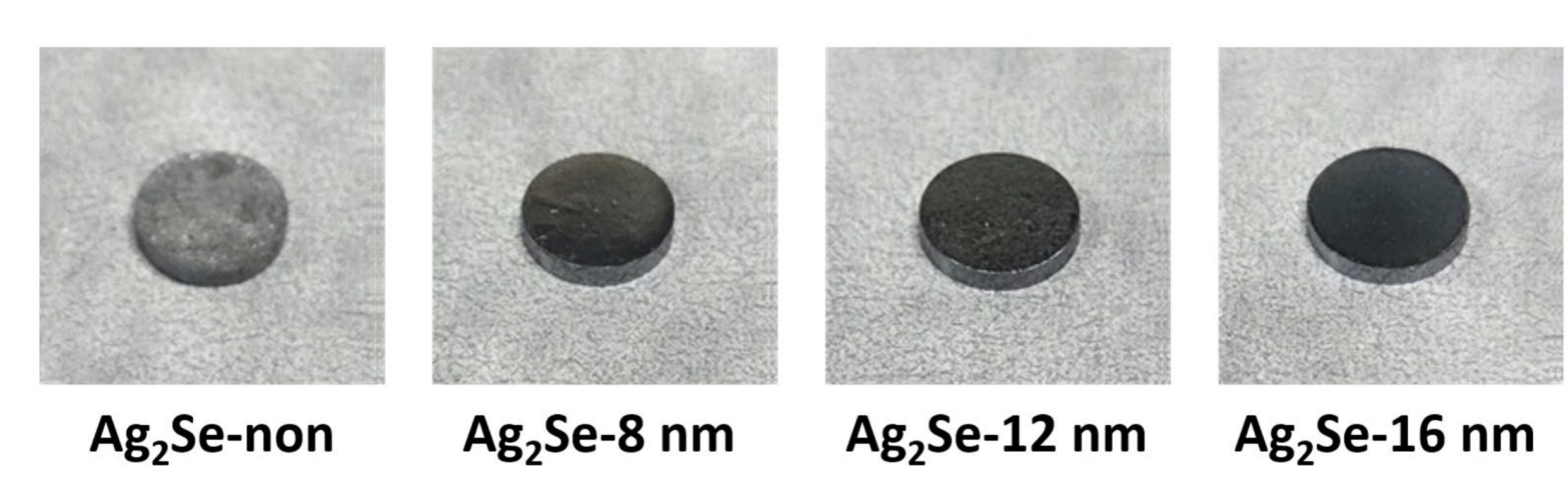

Figure S15. Photographs of pellet samples for thermoelectric measurements prepared by compression at room temperature.

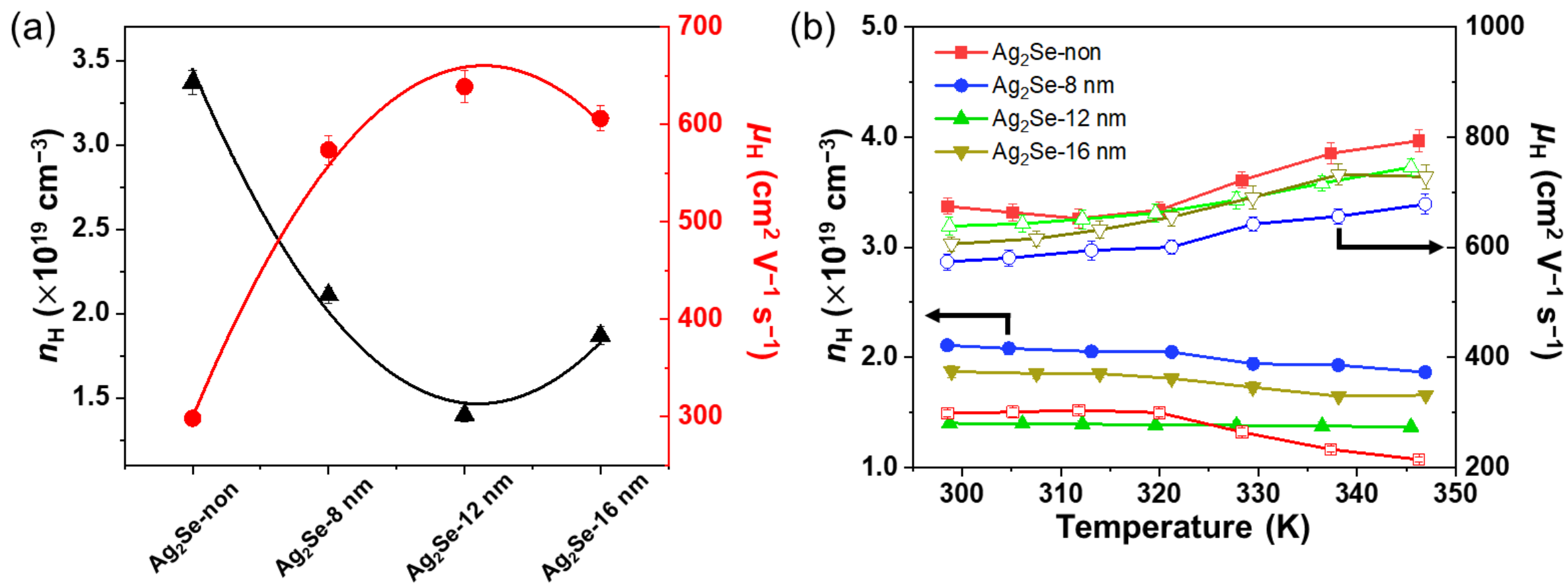

Figure S16. (a) Hall carrier concentration (n_H) and Hall mobility (μ_H) of the Ag_2Se samples at room temperature. (b) Temperature-dependent n_H (filled symbols) and μ_H (open symbols) of the Ag_2Se samples.

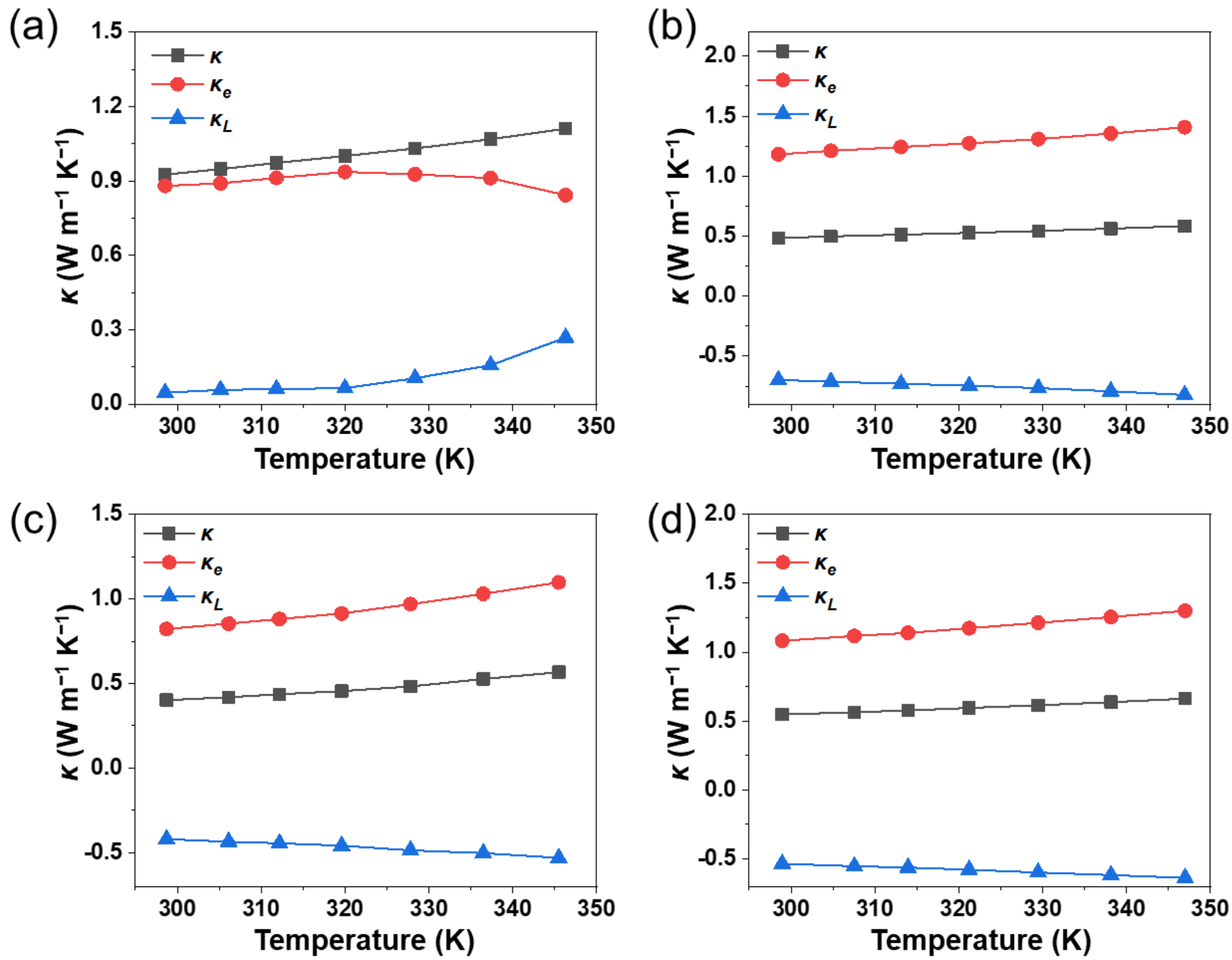

Figure S17. Measured total thermal conductivity (κ), together with the electronic thermal conductivity (κ_e) and lattice thermal conductivity (κ_L) estimated using the Wiedemann–Franz relationship, for (a) Ag_2Se-non, (b) Ag_2Se-8 nm, (c) Ag_2Se-12 nm, and (d) Ag_2Se-16 nm. The Lorenz number was estimated using the Kim–Snyder equation.[15]

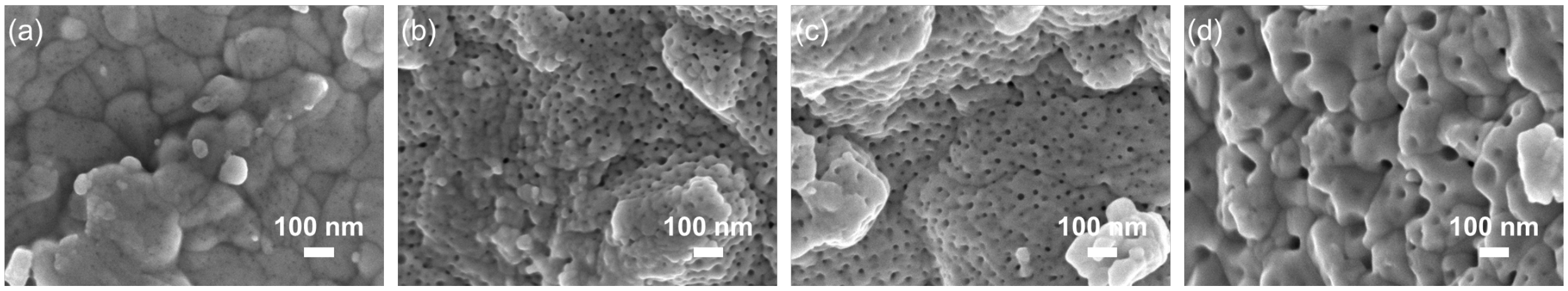

Figure S18. (a-c) SEM images of the pellets crushed after thermoelectric measurements: (a) Ag_2Se-8 nm, (b) Ag_2Se-12 nm, and (c) Ag_2Se-16 nm. (d) SEM image of a highly compressed region in the Ag_2Se-12 nm pellet, showing partial pore deformation and coalescence.

Table S1. Solution compositions and experimental results of zeta potential measurements.

**Sample**	PS-b-PEO in DMF	EtOH	H_2O	0.3 M HNO_3	8 mM $AgNO_3$	8 mM $NaNO_3$	8 mM SeO_2	Total volume	**Zeta potential**	**Mobility**	**Conductivity**
	mL								mV	$cm^2\ V^{-1}\ s^{-1}$	$mS\ cm^{-1}$
**1**	1.5	0.5	3					5	−0.585	−2.19E−06	0.046
**2**	1.5	0.5	1	2				5	−8.62	−3.22E−05	21.10
**3**	1.5	0.5	0.5	2	0.5			5	−13.42	−5.01E−05	12.94
**4**	1.5	0.5	0.5	2		0.5		5	−10.32	−3.85E−05	20.20
**5**	1.5	0.5	0.5	2			0.5	5	−19.20	−7.17E−05	21.083
**6**	1.5	0.5		2	0.5		0.5	5	−14.93	−5.57E−05	14.90

Table S2. Comparison of the near-room-temperature *zT* and thermal conductivity values of bulk-type Ag_2Se reported in the literature with Ag_2Se-12 nm.

Sample	T (K)	κ (W m^{-1} K^{-1})	S (μV K^{-1})	σ (S cm^{-1})	$S^2\sigma$ (μW cm^{-1} K^{-2})	zT	Ref
**β-Ag_2Se**	300	0.75	−150	800	~18.0	0.7	16
**$Ag_2Se_{1.01}$**	300	1.0	−140	-	~32.0	0.9	17
**$Ag_2Se_{1.02}$**	300	1.1	−140	1500	~26.0	0.7	18
**Ag_2Se-ZM**	300	0.81	−150	1000	~20.0	0.75	19
**Ag_2Se/CNTs-0.5**	300	0.85	−160	870	~23.0	0.8	20
**Ag_2Se**	310	0.79	−150	1000	22.5	0.9	21
**β-Ag_2Se**	300	1.0	−120	1500	~22.5	0.65	22
**Ag_2Se**	300	0.98	−160	1160	31.2	0.95	23
**Ag_2Se-135**	320	0.9	−175	1000	~31.5	1.1	24
**Ag_2Se-7% CdSe**	300	1.0	−169	1200	34.0	1.0	25
**$Ag_{1.9}Pb_{0.01}Se$**	300	~0.6	−160	750	~19.0	0.95	26
**Ag_2Se-30%NS**	307	0.73	−145	1200	~24.3	1.04	27
**Ag_2Se-12 nm (This Work)**	298	0.40	−96	1420	13.1	0.98	-

References

1. G. Kresse and J. Furthmüller, *Comput. Mater. Sci.*, 1996, **6**, 15-50.
2. G. Kresse and D. Joubert, *Phys. Rev. B*, 1999, **59**, 1758-1775.
3. P. E. Blöchl, *Phys. Rev. B*, 1994, **50**, 17953-17979.
4. S. Grimme, *WIREs Comput. Mol. Sci.*, 2011, **1**, 211-228.
5. S. Grimme, S. Ehrlich and L. Goerigk, *J. Comput. Chem.*, 2011, **32**, 1456-1465.
6. I. A. Balyakin and S. I. Sadovnikov, *Comput. Mater. Sci.*, 2022, **202**, 110963.
7. R. Jacobs, D. Morgan, S. Attarian, J. Meng, C. Shen, Z. Wu, C. Y. Xie, J. H. Yang, N. Artrith, B. Blaiszik, G. Ceder, K. Choudhary, G. Csanyi, E. D. Cubuk, B. Deng, R. Drautz, X. Fu, J. Godwin, V. Honavar, O. Isayev, A. Johansson, B. Kozinsky, S. Martiniani, S. P. Ong, I. Poltavsky, K. J. Schmidt, S. Takamoto, A. P. Thompson, J. Westermayr and B. M. Wood, *Curr. Opin. Solid State Mater. Sci.*, 2025, **35**, 101214.
8. A. Hjorth Larsen, J. Jørgen Mortensen, J. Blomqvist, I. E. Castelli, R. Christensen, M. Dułak, J. Friis, M. N. Groves, B. Hammer, C. Hargus, E. D. Hermes, P. C. Jennings, P. Bjerre Jensen, J. Kermode, J. R. Kitchin, E. Leonhard Kolsbjerg, J. Kubal, K. Kaasbjerg, S. Lysgaard, J. Bergmann Maronsson, T. Maxson, T. Olsen, L. Pastewka, A. Peterson, C. Rostgaard, J. Schiøtz, O. Schütt, M. Strange, K. S. Thygesen, T. Vegge, L. Vilhelmsen, M. Walter, Z. Zeng and K. W. Jacobsen, *J. Phys.: Condens. Matter*, 2017, **29**, 273002.
9. J. Zeng, D. Zhang, D. Lu, P. Mo, Z. Li, Y. Chen, M. Rynik, L. A. Huang, Z. Li, S. Shi, Y. Wang, H. Ye, P. Tuo, J. Yang, Y. Ding, Y. Li, D. Tisi, Q. Zeng, H. Bao, Y. Xia, J. Huang, K. Muraoka, Y. Wang, J. Chang, F. Yuan, S. L. Bore, C. Cai, Y. Lin, B. Wang, J. Xu, J.-X. Zhu, C. Luo, Y. Zhang, R. E. A. Goodall, W. Liang, A. K. Singh, S. Yao, J. Zhang, R. Wentzcovitch, J. Han, J. Liu, W. Jia, D. M. York, W. E, R. Car, L. Zhang and H. Wang, *J. Chem. Phys.*, 2023, **159**, 054801.
10. H. Wang, L. Zhang, J. Han and W. E, *Comput. Phys. Commun.*, 2018, **228**, 178-184.
11. L. Zhang, J. Han, H. Wang, W. Saidi, R. Car and W. E, End-to-end symmetry preserving inter-atomic potential energy model for finite and extended systems, in *Advances in Neural Information Processing Systems* 31 (NeurIPS 2018).
12. A. P. Thompson, H. M. Aktulga, R. Berger, D. S. Bolintineanu, W. M. Brown, P. S. Crozier, P. J. in 't Veld, A. Kohlmeyer, S. G. Moore, T. D. Nguyen, R. Shan, M. J. Stevens, J. Tranchida, C. Trott and S. J. Plimpton, *Comput. Phys. Commun.*, 2022, **271**, 108171.
13. F. Müller-Plathe, *J. Chem. Phys.*, 1997, **106**, 6082-6085.
14. T. Ikeshoji and B. Hafskjold, *Mol. Phys.*, 1994, **81**, 251-261.
15. H.-S. Kim, Z. M. Gibbs, Y. Tang, H. Wang and G. J. Snyder, *APL Mater.*, 2015, 3, 041506
16. J. Chen, Q. Sun, D. Bao, T. Liu, W.-D. Liu, C. Liu, J. Tang, D. Zhou, L. Yang and Z.-G. Chen, *ACS Appl. Mater. Interfaces*, 2020, **12**, 51523-51529.
17. P. Jood, R. Chetty and M. Ohta, *J. Mater. Chem. A*, 2020, **8**, 13024-13037.
18. S. Huang, T.-R. Wei, H. Chen, J. Xiao, M. Zhu, K. Zhao and X. Shi, *ACS Appl. Mater. Interfaces*, 2021, **13**, 60192-60199.
19. M. Jin, J. Liang, P. Qiu, H. Huang, Z. Yue, L. Zhou, R. Li, L. Chen and X. Shi, *J. Phys. Chem. Lett.*, 2021, **12**, 8246-8255.
20. H. Wang, X. Liu, Z. Zhou, H. Wu, Y. Chen, B. Zhang, G. Wang, X. Zhou and G. Han, *Acta Mater.*, 2022, **223**, 117502.
21. D. Palaporn, K. Kurosaki and S. Pinitsoontorn, *Adv. Energy Sustainability Res.*, 2023, **4**, 2300082.
22. S. Lin, L. Guo, X. Wang, Y. Liu, Y. Wu, R. Li, H. Shao and M. Jin, *J. Materiomics*, 2023, **9**, 754-761.
23. F. Jiang, C. Lin, J. Cheng, H. Yu, Y. Zhou, X. Ma, L. Wu, S. Ye, J. Chen, S. Zhi, Y. Xu, P. Zhao, X. Wang, F. Cao, Q. Zhang and J. Mao, *Adv. Funct. Mater.*, 2025, **35**, 2415000.
24. T. Kleinhanns, F. Milillo, M. Calcabrini, C. Fiedler, S. Horta, D. Balazs, M. J. Strumolo, R. Hasler, J. Llorca, M. Tkadletz, R. L. Brutchey and M. Ibáñez, *Adv. Energy Mater.*, 2024, **14**, 2400408.
25. Y. Liu, T. Kleinhanns, S. Horta, E. P. Dutkiewicz-Kopczynska, S. Lu, M. C. Spadaro, A. Genç, L. Chen, K. H. Lim, M. Hong, J. Arbiol and M. Ibáñez, *J. Am. Chem. Soc.*, 2025, **147**, 32199-32208.
26. K. H. Lim, Y. Xia, L. Xu, M. Zhao, M. Li, Y. Cheng, J. Mao, S. Wang, L. Chen, S. W. Tsang, P. Liu, Q. Wang, X. Yang, W.-J. Wang, A. Cabot, M. Hong, Y. Zhang and Y. Liu, *Chem. Eng. J.*, 2025, **511**, 162265.
27. P. Kitthonbancha, W.-D. Liu, M. Li, S. Pinitsoontorn and Z.-G. Chen, *Small*, 2025, **21**, 2411798.
